# Extracellular loops 2 and 3 of the calcitonin receptor selectively modify agonist binding and efficacy

**DOI:** 10.1016/j.bcp.2018.02.005

**Published:** 2018-04

**Authors:** Emma Dal Maso, Yue Zhu, Vi Pham, Christopher A. Reynolds, Giuseppe Deganutti, Caroline A. Hick, Dehua Yang, Arthur Christopoulos, Debbie L. Hay, Ming-Wei Wang, Patrick M. Sexton, Sebastian G.B. Furness, Denise Wootten

**Affiliations:** a Drug Discovery Biology, Monash Institute of Pharmaceutical Sciences, Monash University, Parkville 3052, Victoria, Australia; b The National Center for Drug Screening and CAS Key Laboratory of Receptor Research, Shanghai Institute of Materia Medica, Chinese Academy of Sciences, Shanghai 201203, China; c School of Biological Sciences, University of Essex, Wivenhoe Park, Colchester CO4 3SQ, United Kingdom; d The University of Auckland, School of Biological Sciences, 3 Symonds Street, Auckland 1142, New Zealand; e University of Chinese Academy of Sciences, Beijing 100049, China; f School of Pharmacy, Fudan University, Shanghai 201203, China; g University of Chinese Academy of Sciences, 19A Yuquan Road, Beijing 100049, China

**Keywords:** G protein-coupled receptor, Calcitonin receptor, GPCR structure-function, Biased agonism, Molecular modelling

## Abstract

Class B peptide hormone GPCRs are targets for the treatment of major chronic disease. Peptide ligands of these receptors display biased agonism and this may provide future therapeutic advantage. Recent active structures of the calcitonin (CT) and glucagon-like peptide-1 (GLP-1) receptors reveal distinct engagement of peptides with extracellular loops (ECLs) 2 and 3, and mutagenesis of the GLP-1R has implicated these loops in dynamics of receptor activation. In the current study, we have mutated ECLs 2 and 3 of the human CT receptor (CTR), to interrogate receptor expression, peptide affinity and efficacy. Integration of these data with insights from the CTR and GLP-1R active structures, revealed marked diversity in mechanisms of peptide engagement and receptor activation between the CTR and GLP-1R. While the CTR ECL2 played a key role in conformational propagation linked to Gs/cAMP signalling this was mechanistically distinct from that of GLP-1R ECL2. Moreover, ECL3 was a hotspot for distinct ligand- and pathway-specific effects, and this has implications for the future design of biased agonists of class B GPCRs.

## Graphical abstract

**Figure:**
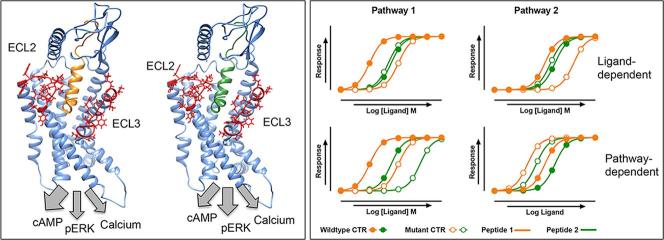

## 1 Introduction

Class B1 G protein-coupled receptors (GPCRs) are the targets for peptide hormones that play major roles in the development and maintenance of lymphatic and cardiovascular function, bone homeostasis, metabolic regulation, migraine, stress and anxiety [1]. Consequently, these receptors are important therapeutic targets.

The calcitonin (CT), Class B1 GPCRs (CTRs), are highly expressed on osteoclasts and have been exploited therapeutically for treatment of bone disorders, including Paget’s disease, hypercalcemia of malignancy and osteoporosis [2], [3], [4], [5]. The receptors are also expressed in numerous other cells and tissues including leucocytes and their precursors, the central nervous system, kidney, lung, gastrointestinal tract and reproductive tissues [6], thereby influencing pain perception, feeding and reproduction, and ion secretion [2], [7], though these actions are not well understood. Furthermore, CTRs can interact with the receptor activity modifying protein (RAMP) family to form high affinity receptors for amylin (Amy) and calcitonin gene-related peptide (CGRP) [8].

CT peptides from different species have been identified and can be classified into 3 major subgroups based on evolution and sequence conservation: human/rodent, artiodactyl (e.g., porcine) and teleost (salmon/eel)/chicken. Both human and salmon CT have been approved for treatment of bone disorders including Paget’s disease and osteoporosis, however, they have distinct binding kinetics, affinity and efficacy [9], [10], [11] that impact on G protein recruitment and activation [11], suggesting different modes of interaction with CTRs.

Orthosteric peptide ligands of Class B1 GPCRs are proposed to interact with their cognate receptors via a two-domain mechanism, with an initial engagement of the C-terminus of the peptide with the N-terminal extracellular domain (ECD) of the receptor that allows the peptide N-terminus to bind to the transmembrane (TM) spanning receptor core comprising the 7 TM helices and 3 interconnecting extracellular loops (ECLs), leading to receptor activation [12]. This mode of binding is supported by recent full-length, active, Gs-complexed structures of the CTR [13] (bound to sCT) and glucagon-like peptide-1 (GLP-1) receptor (GLP-1R) (bound to GLP-1 [14], or exendin-P5 [15]). Nonetheless, there were marked differences in the orientation of the receptor ECD relative to the receptor core and correlative changes in presentation of the peptides to the receptor core, linked to differences in the degree of secondary structure of the peptides [13], [14], [15]. Moreover, the CT-family peptides have a cyclic, cysteine-disulfide linked N-terminus between amino acids 1 and 7 (2 and 7 for Amy and CGRP) that contrasts with the extended helix of GLP-1, and alters the relative interaction of the peptide N-termini with the ECLs and proximal TM helix segments.

Mutagenesis and crosslinking studies have shown that the ECLs of Class B1 GPCRs are critical for both peptide binding and propagation of conformational change associated with receptor activation [14], [15], [16], [17], [18], [19], [20]. For the GLP-1R, alanine-scanning mutagenesis revealed that the ECLs, particularly ECL2 and ECL3, were also important in the biased agonism of peptides, but had distinct contribution to pathway specific signalling [16], [21]. For this receptor, both ECL2 and ECL3 played a critical role in cAMP formation, and intracellular calcium (_i_Ca^2+^) mobilisation, while effects on ERK phosphorylation (pERK) were principally confined to residues within ECL3.

In the current study, we have performed alanine-scanning mutagenesis of amino acids in ECLs 2 and 3 of the hCTR and interrogated mutant receptors for their effects on cell surface receptor expression, peptide affinity and efficacy for cAMP and IP1 accumulation, as well as pERK in response to calcitonin (sCT, hCT, pCT) and related family (Amy, CGRP) peptides. This work revealed both differences in how the receptor engages with and is activated by the different CT-family peptides, and in the role of ECLs 2 and 3 between the CTR and GLP-1R.

## 2 Materials and methods

### 2.1 Reagents

All peptides were purchased from Mimotopes. Dulbecco’s Modified Eagle’s Medium (DMEM) was purchased from Invitrogen. Foetal bovine serum (FBS) was purchased from Thermo Electron Corporation. AlphaScreen reagents, Lance cAMP kit, 384-well Optiplates were purchased from PerkinElmer. SureFire™ ERK1/2 reagents were obtained from TGR Biosciences and PerkinElmer. IP-One HTRF® assay kit was from CisBio. Antibodies were purchased from R&D systems and ThermoFisher. All other reagents were purchased from Sigma-Aldrich or BDH Merck and were of an analytical grade.

### 2.2 Mutagenesis

Desired mutations were introduced to N-terminally c-Myc tagged human CTR in pENTER11 (Invitrogen) via the Q5® High-Fidelity PCR Kit (New England Biolabs), then LR recombination reactions were conducted to transfer mutated and wild-type (WT) receptor into the pEF5/FRT/V5-DEST destination vector using Gateway Technology (Invitrogen). The oligonucleotides for mutagenesis were purchased from Gene-Works (Thebarton, SA, Australia) and mutants were confirmed by automated-sequencing.

### 2.3 Stable cell line generation and cell culture

The mutant or wild-type (WT) receptor genes were integrated into FlpIn-CV1 cells using Flp-InTM system (Invitrogen). Stable Flp-In expression cell lines were generated through polyclonal selection, screening and maintained in Dulbecco’s modified Eagle’s medium supplemented with 5% (v/v) FBS, 300 μg/ml hygromycin B (Invitrogen) at 37 °C in 5% CO_2_.

### 2.4 Homologous whole cell competition binding assay

Homologous radioligand competition was performed on 2.5 × 10^4^ cells/well seeded into 96-well trays and cultured overnight. Cells were incubated overnight at 4 °C with two concentrations of ^125^I-sCT(8-32) (between 25 and 100 pM; specific activity, 2000 Ci/mmol^9^) and serial dilutions of non-iodinated sCT(8-32). Non-bound ligand was removed and bound ligand activity was measured using a γ counter (Wallac Wizard 1470 Gamma Counter, Perkin Elmer, 80% counter efficiency). Values were normalized against non-specific binding (0%) defined by the presence of 1 μM of unlabelled sCT(8-32), and total ligand bound in absence of competing sCT(8-32) (100%).

### 2.5 Heterologous whole cell competition binding assay

Competition binding was performed as previously described [41] on whole cells in 96-well plates by using the antagonist radioligand ^125^I-sCT(8-32) (∼0.1 nM), and competing with increasing concentrations of unlabelled peptide. Non-specific binding was defined by co-incubation with 1 μM unlabelled sCT(8-32). Following overnight incubation non-bound ligand was removed and radioactivity was determined using a gamma counter.

### 2.6 Cell surface expression by FACS

Surface expression of c-Myc tagged CTR mutants stably expressed in CV-1 cells was quantified by flow cytometry using standard methods. Cells were plated into 6 well trays at approximately 5 × 10^5^ cells per well the day before assay. Cells were harvested using versene. All staining steps were conducted in ice cold HBSS with 0.1% BSA and 20 mM HEPES (pH 7.4). Blocking was conducted in 5% BSA. Primary antibody staining was performed with a mixture of 5 µg/mL 9E10 (anti-c-Myc) and 1 µg/mL Mab4614 (anti-CTR, R&D Systems). The secondary antibody was 1 µg/mL goat anti-mouse AF647 (ThermoFisher). Sytox blue was used for live/dead discrimination. Data were collected on a FACS CantoII (BD Biosciences) with at least 20,000 live cells collected per sample, WT stained CTR sample and stained parental CV-1 cells were collected at the beginning and the end of each run. Data were analysed using FlowJo. The mean AF647 fluorescence intensity from each sample for a particular experiment was normalized against parental (0%) and CTR WT (100%) controls.

### 2.7 cAMP accumulation

Cells (2.5 × 10^4^ cells/well) were seeded into 96-well plates and incubated overnight. Cells were stimulated with increasing concentrations of ligands for 30 min in the presence of IBMX. The liquid was discarded, changed to absolute ethanol and volatilized to dryness at room temperature. Samples were then lysed and intracellular cAMP was detected using PerkinElmer Lance kit as previously described [16]. Data were normalized to the response of 100 μM forskolin.

### 2.8 IP1 accumulation

Cells (2.5 × 10^4^ cells/well) were plated into 96 well trays and cultured overnight. Cells were stimulated with increasing concentration of ligands for 60 min in the presence of LiCl. Samples were lysed and endogenous IP_1_ was measured using an IP-One HTRF® assay kit (CisBio) as specified by the manufacturer. Data were normalized to the maximal response elicited by 100 μM of ATP.

### 2.9 ERK1/2 phosphorylation

Cells (2.5 × 10^4^ cells/well) were seeded into 96-well culture plates and incubated overnight. Initially, pERK1/2 time-course experiments were performed over 30 min to identify the time point when the pERK1/2 response is maximal (6–8 min). Subsequently, this time point was selected to generate concentration response for different agonists with ligand addition performed after overnight serum starvation. pERK1/2 was detected using an Alphascreen assay as previously described [22]. Data were normalized to the maximal response elicited by 10% FBS determined at 6 min.

### 2.10 Data analysis

IC_50_ and Bmax values were estimated from competitive inhibition of ^125^I-sCT(8-32) binding using a 3-parameter logistic equation (log(inhibitor versus response)) in Prism (v7; Graphpad). Data were corrected for radioligand occupancy using the Cheng-Prusoff equation in Prism; as such data are reported as pK_i_. Emax and EC_50_ were estimated from concentration-response curves using with a 3-parameter logistic equation in Prism (v7). These values are a composite of functional affinity, efficacy and stimulus response coupling. The Black and Leff operational model of agonism [23] was applied to separate effects on pathway-specific efficacy (τ) from those that modify ligand functional affinity (pK_A_). Derived τ values were normalized to experimentally determined levels of cell surface expression to provide a measure of efficacy (τ_c_) that is independent of affinity and altered cell surface receptor expression [16]. pKi, pK_A_ and log τc values for mutant receptors were statistically compared to those of the WT receptor using a one-way analysis of variance (ANOVA) and Dunnett’s post-test. Significance was accepted at P < 0.05.

### 2.11 Molecular modelling and mapping of mutational effects

The structures of the CTR:CT complexes were generated from the cryo-electron microscopy structure of the calcitonin receptor [13] and the X-ray structure of the CTR ECD [24]. Missing loops were generated using Modeller [25] (1000 structures) and the final loop selected to ensure that the conserved residues faced inwards [26] by analysis of the correlation of the conservation, as measured using the Shannon entropy, and the extent to which a residue is buried [27].

### 2.12 Molecular dynamics simulations

CTR:sCT and CTR:hCT complexes were prepared for molecular dynamics simulations by means of a multistep procedure that integrates both python htmd [28] and tcl (Tool Command Language) scripts. The pdb2pqr [29] and propka [30] software were used to check the protein’s structural integrity and to add hydrogen atoms (configurations of titratable amino acid side chains were visually inspected) appropriate for a simulated pH of 7.0. The CTR was embedded in rectangular matrixes of a 1-palmitoyl-2-oleyl-sn-glycerol-3-phospho-choline (POPC) bilayer (previously built by using the VMD Membrane Builder plugin 1.1; http://www.ks.uiuc.edu/Research/vmd/plugins/membrane) through an insertion method [31]: receptors were first oriented according to the CTR coordinates from the OPM database [32], then lipids overlapping the protein were removed and TIP3P water molecules [33] were added to the simulation box by means of the VMD Solvate plugin 1.5 (http://www.ks.uiuc.edu/Research/vmd/plugins/solvate). Charge neutrality was finally reached by adding Na^+^/Cl^−^ counter ions to a final concentration of 0.154 M, according to the VMD Autoionize plugin 1.3 (http://www.ks.uiuc.edu/Research/vmd/plugins/autoionize). The CHARMM36 force field [34] was used.

### 2.13 System equilibration and classic MD run settings

All the following MD simulation stages were performed by using Acemd [35]. Equilibration of the four systems was achieved in isothermal-isobaric conditions (NPT) using the Berendsen barostat [36] (target pressure 1 atm) and the Langevin thermostat [37] (target temperature 300 K) with a low damping of 1 ps^−1^. A three-stage procedure with an integration time step of 2 fs was performed: in the first stage, 2000 conjugate-gradient minimization steps were applied to reduce the clashes between protein and lipids. Then, a 10 ns long MD simulation was performed in the NPT ensemble, with a positional constraint of 1 kcal mol^−1^ Å^−2^ on protein and lipid phosphorus atoms. During the second stage, 30 ns of MD simulation in the NPT ensemble were performed, constraining all protein atoms but leaving the POPC residues free to diffuse in the bilayer. In the last equilibration stage, positional constraints were reduced by one half and applied only to the protein backbone alpha carbons for a further 10 ns of MD simulation.

For each intermolecular complex, a total of 2 μs of unbiased MD was performed, divided in one replica 1 μs long and two replicas 500 ns long. After equilibration, production MD trajectories were computed with an integration time step of 4 fs in the canonical ensemble (NVT) at 300 K, using a thermostat damping of 0.1 ps^−1^ and the M-SHAKE algorithm [38] to constrain the bond lengths involving hydrogen atoms. The cut off distance for electrostatic interactions was set at 9 Å, with a switching function applied beyond 7.5 Å. Long range Coulomb interactions were handled using the particle mesh Ewald summation method (PME) [39] by setting the mesh spacing to 1.0 Å

### 2.14 Analysis

Contacts and hydrogen bonds were quantified using VMD [40]. A contact between two residues was considered productive if at least two atoms were detected at distances less than 3.5 Å. A distance between acceptor and donor atoms of 3 Å and an angle value of 20° were set as the geometrical cut-off for hydrogen bonds. Equilibrated coordinates and parameters are available from the following doi: https://doi.org//10.5526/ERDR-00000075.

Data on the effect of ECL2 and ECL3 mutation on GLP-1R-mediated efficacy were mapped onto the high resolution 3.3A structure of exendin-P5 in complex with the hGLP-1R and dominant negative Gs heterotrimer (PDB = 6B3J [15]). Mapping and visualization of the effect of mutation on receptor structure was performed using ICM (Molsoft).

## 3 Results

**Fig. 1:**
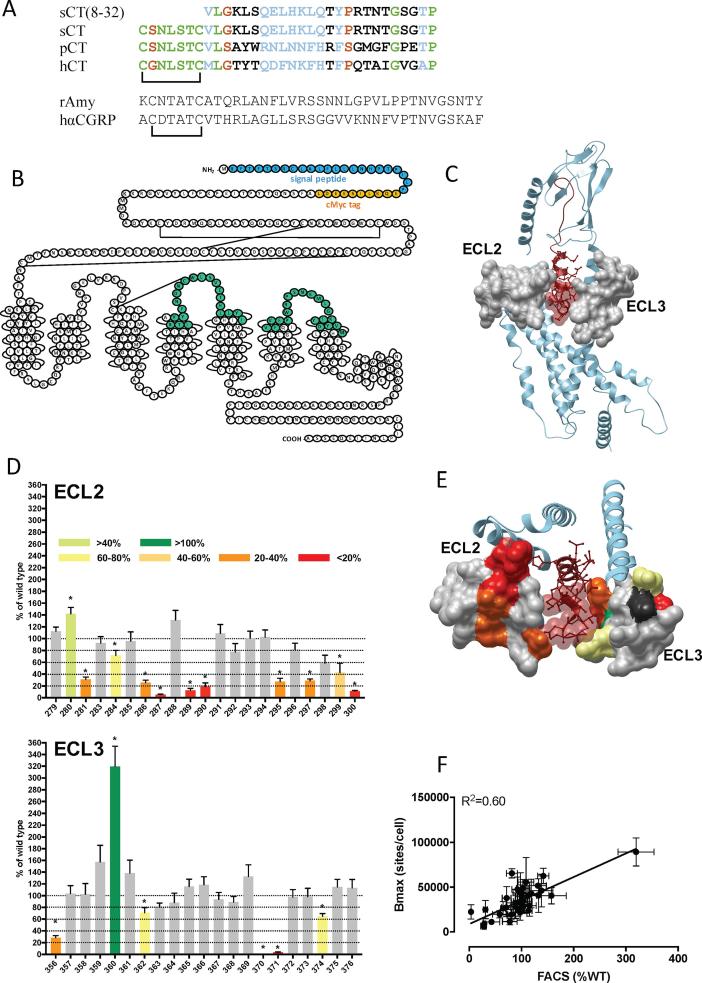
Alanine mutation of ECL2 and ECL3 of the hCTR selectively alters expression of the receptor. (A) Alignment of CT and related peptide sequences was performed using Biology WorkBench (workbench.sdsc.edu). Identical residues are highlighted in green, conservative substitutions are coloured blue, and semi- conservative substitutions are in orange. Black text indicates the non-conserved. (B) Snake diagram of the hCTR: highlighted in blue are the residues that constitute the signal peptide of the receptor, in orange the c-Myc tag, and in green the residues that have been mutated to alanine. (C) Active state model of the hCTR (pale blue ribbon), with position of mutated residues displayed as grey surface map. sCT is shown as dark red, with side chains in proximity to the ECLs displayed in x-stick, and residues 1–7 that are critical for receptor activation displayed in transparent cpk. (D) Expression of mutant receptors determined by FACS analysis of antibody binding to the c-Myc epitope. (E) Top view of the ECLs with mutants that significantly altered receptor expression displayed in colour according to the magnitude of effect; grey indicates no significant effect and black mutants where expression could not be measured. The receptor ECD and C-terminal peptide residues are omitted for clarity (F). There was a strong correlation between cell surface receptor expression by FACS and homologous competition radioligand binding. ^*^P < 0.05, one-way ANOVA with Dunnett’s post-test. Data are mean + S.E.M. (D) or ±S.E.M. (F) of 3–6 (WT 10–12) independent experiments. (For interpretation of the references to colour in this figure legend, the reader is referred to the web version of this article.)

To assess the importance of ECL2 and ECL3 in CTR function, we performed alanine-scanning mutagenesis of residues within these loops as well as adjacent TMs, and assessed effects on cell surface receptor expression, competitive binding affinity and cAMP accumulation, IP1 accumulation and ERK1/2 phosphorylation (pERK). These pathways are important for physiological, CTR-mediated, signalling [2], [7]. Responses were evaluated for representatives of the major structural/evolutionary clades of CT peptides, specifically, human CT (hCT) and salmon CT (sCT) that are both used clinically, as well as porcine CT (pCT) (Fig. 1A). We also assessed responses to the related peptides Amy and CGRP that bind to and activate the CTR with low affinity/potency, but are potent agonists of CTR/RAMP complexed receptors. Global affinity (pK_i_) was determined from competition with the radiolabelled antagonist peptide ^125^I-sCT(8-32), while functional affinity (pK_A_) and efficacy were determined by quantification of concentration-response data with the operational model of Black and Leff [23] that provides independent measures of pK_A_ and efficacy (τ). All experiments were performed in CV-1 FlpIn cells that lack functional CTR and RAMP expression, with receptors stably expressed following isogenic recombination. Data are mapped onto the active hCTR structure (5UZ7), following modelling of missing side chains and sampling by short time-scale MD.

### 3.1 Receptor expression

Residues for mutation were selected based on the recent structure of sCT/hCTR/Gs [13] and comprised amino acids I279-I300 (ECL2) and F356-M376 (ECL3) (Fig. 1B,C). Cell surface expression was determined by anti-c-Myc antibody binding to the c-Myc tag inserted after the receptor signal peptide in the N-terminal ECD (Fig. 1B), and quantified by FACS.

Most mutants demonstrated equivalent cell surface expression to that of the WT receptor (Fig. 1D; Table 1), however, a subset of mutants, particularly within ECL2, had altered expression. Only K370A in ECL3 had no detectable expression. D287A, C289A, W290A, and I300A in ECL2, and I371 in ECL3, also had markedly diminished cell surface expression (<20% of WT). R281A, N286A, T295A, L297A, Y299A in ECL2 and F356A in ECL3 were expressed at levels between 20% and ∼40% of WT. A smaller but significant attenuation of expression was observed for Y284A, R362A and Y374A. In contrast, T280A significantly increased expression and P360A strongly augmented cell surface receptor expression (Fig. 1D; Table 1). Within ECL2, those residues with strongly attenuated expression formed a continuum within the central portion of the loop, suggesting that these residues participate in a network that helps to stabilize the apo receptor (Fig. 1E).

**Table 1:** Effect of single alanine mutation in hCTR ECL2 or ECL3 on binding affinity (pK_i_) of CT peptides, and receptor cell surface expression.

	hCT	sCT	pCT	sCT(8-32)	FACS	Bmax
	pK_i_	pK_i_	pK_i_	pK_i_	(% of WT)	(sites/cell)
WT	6.72 ± 0.06 (16)	9.87 ± 0.03 (16)	8.27 ± 0.07 (17)	9.70 ± 0.05 (18)	100 (12)	22,900 ± 2500 (10)
I279A	6.16 ± 0.08 (3)	9.93 ± 0.18 (4)	7.83 ± 0.24 (4)	9.72 ± 0.08 (8)	113 ± 7 (4)	40,500 ± 11,300 (5)
T280A	6.71 ± 0.05 (4)	10.2 ± 0.03 (4)	8.20 ± 0.15 (4)	9.61 ± 0.08 (8)	142 ± 11 (4)	62,600 ± 8700^*^ (4)
R281A	5.92 ± 0.28^*^ (5)	9.10 ± 0.15^*^ (6)	7.18 ± 0.23^*^ (5)	9.07 ± 0.21^*^ (10)	32 ± 4^*^ (6)	24,900 ± 10,200 (3)
V283A	6.63 ± 0.11 (4)	9.78 ± 0.14 (4)	7.82 ± 0.08 (4)	9.67 ± 0.03 (8)	93 ± 11 (4)	47,600 ± 13,600 (4)
Y284A	6.61 ± 0.11 (5)	9.83 ± 0.07 (3)	7.84 ± 0.10 (4)	9.85 ± 0.10 (8)	72 ± 9^*^ (4)	37,800 ± 12,800 (4)
F285A	6.60 ± 0.11 (4)	10.0 ± 0.12 (4)	7.92 ± 0.11 (4)	9.99 ± 0.05 (8)	96 ± 15 (4)	33,200 ± 11,000 (4)
N286A	N.D.	N.D.	N.D.	N.D.	26 ± 4^*^ (4)	N.D.
D287A	N.D.	N.D.	N.D.	N.D.	5 ± 1^*^ (4)	N.D.
N288A	6.35 ± 0.12 (4)	9.74 ± 0.13 (4)	8.03 ± 0.12 (5)	9.63 ± 0.04 (8)	132 ± 16 (4)	51,600 ± 2600^*^ (4)
C289A	N.D.	N.D.	N.D.	N.D.	13 ± 3^*^ (3)	N.D.
W290A	N.D.	N.D.	N.D.	N.D.	19 ± 6^*^ (4)	N.D.
L291A	5.41 ± 0.10^*^ (3)	9.75 ± 0.21 (5)	7.17 ± 0.12^*^ (6)	8.76 ± 0.19^*^ (9)	109 ± 15 (4)	55,700 ± 27,400 (3)
S292A	6.60 ± 0.05 (4)	10.4 ± 0.20^*^ (4)	8.54 ± 0.22 (4)	10.2 ± 0.13 (8)	78 ± 14 (4)	11,600 ± 3600 (4)
V293A	6.22 ± 0.08 (4)	9.59 ± 0.05 (4)	7.85 ± 0.10 (4)	9.75 ± 0.08 (8)	101 ± 12 (4)	39,500 ± 13,000 (4)
E294A	6.73 ± 0.06 (5)	9.77 ± 0.08 (4)	7.93 ± 0.10 (4)	9.58 ± 0.07 (8)	103 ± 12 (4)	48,700 ± 10,100 (4)
T295A	5.90 ± 0.050^*^ (4)	10.2 ± 0.16 (4)	7.49 ± 0.08^*^ (4)	10.3 ± 0.09^*^ (8)	27 ± 6^*^ (4)	6600 ± 2300^*^ (4)
H296A	6.95 ± 0.13 (4)	9.84 ± 0.09 (4)	8.07 ± 0.03 (4)	9.57 ± 0.07 (8)	82 ± 11 (4)	65,500 ± 5100^*^ (4)
L297A	6.09 ± 0.05^*^ (4)	10.1 ± 0.15 (4)	7.81 ± 0.19 (4)	10.2 ± 0.07 (8)	29 ± 3^*^ (4)	9200 ± 2000^*^ (4)
L298A	6.13 ± 0.08^*^ (4)	9.76 ± 0.09 (4)	7.69 ± 0.10 (4)	9.83 ± 0.12 (8)	59 ± 14 (4)	19,600 ± 4000 (4)
Y299A	6.28 ± 0.02 (4)	9.41 ± 0.20 (4)	7.33 ± 0.20^*^ (4)	9.97 ± 0.18 (8)	43 ± 16^*^ (4)	11,100 ± 1900^*^ (4)
I300A	N.D.	N.D.	N.D.	N.D.	11 ± 1^*^ (4)	N.D.
F356A	6.58 ± 0.11 (5)	9.79 ± 0.34 (5)	7.92 ± 0.17 (4)	9.85 ± 0.25 (12)	28 ± 4^*^ (6)	5900 ± 2200^*^ (5)
V357A	6.13 ± 0.13^*^ (5)	9.83 ± 0.18 (4)	7.89 ± 0.27 (4)	9.68 ± 0.10 (8)	104 ± 14 (4)	28,400 ± 7700 (4)
V358A	5.89 ± 0.15^*^ (5)	9.62 ± 0.09 (4)	7.84 ± 0.13 (4)	9.81 ± 0.06 (8)	103 ± 18 (4)	34,100 ± 10,500 (4)
F359A	5.49 ± 0.12^*^ (5)	9.50 ± 0.15 (4)	7.13 ± 0.19^*^ (3)	9.66 ± 0.10 (10)	158 ± 28 (4)	40,400 ± 9200 (4)
P360A	5.65 ± 0.06^*^ (5)	9.01 ± 0.13^*^ (4)	7.30 ± 0.28^*^ (4)	9.22 ± 0.10^*^ (10)	320 ± 35^*^ (5)	89,400 ± 15,800^*^ (5)
W361A	5.99 ± 0.08^*^ (5)	9.48 ± 0.08 (4)	7.47 ± 0.24^*^ (4)	9.55 ± 0.11 (10)	139 ± 22 (4)	46,200 ± 18,200 (5)
R362A	5.96 ± 0.06^*^ (4)	9.53 ± 0.13 (4)	7.33 ± 0.22^*^ (4)	9.66 ± 0.14 (8)	71 ± 9^*^ (4)	27,300 ± 1000 (4)
P363A	5.48 ± 0.26^*^ (4)	9.44 ± 0.14 (4)	7.26 ± 0.13^*^ (4)	9.45 ± 0.23 (8)	81 ± 7 (4)	19,400 ± 5800 (4)
S364A	6.61 ± 0.07 (4)	9.73 ± 0.06 (4)	8.27 ± 0.34 (4)	9.72 ± 0.08 (10)	88 ± 16 (4)	32,600 ± 11,200 (5)
N365A	6.68 ± 0.16 (4)	9.96 ± 0.06 (4)	8.68 ± 0.24 (4)	9.63 ± 0.10 (10)	116 ± 13 (4)	29,400 ± 11,900 (5)
K366A	6.47 ± 0.17 (4)	9.69 ± 0.05 (4)	7.96 ± 0.23 (4)	9.71 ± 0.12 (10)	119 ± 14 (4)	43,000 ± 16,000 (5)
M367A	6.24 ± 0.11 (4)	9.67 ± 0.09 (4)	7.67 ± 0.25 (4)	9.53 ± 0.14 (8)	94 ± 112 (4)	25,600 ± 7900 (4)
L368A	6.50 ± 0.13 (4)	9.81 ± 0.04 (4)	8.07 ± 0.19 (4)	9.68 ± 0.11 (8)	89 ± 9 (4)	28,200 ± 13,100 (4)
G369A	6.41 ± 0.10 (4)	9.89 ± 0.02 (4)	8.10 ± 0.19 (4)	9.64 ± 0.14 (10)	133 ± 20 (4)	40,600 ± 18,800 (5)
K370A	N.D.	N.D.	N.D.	N.D.	N.D.	N.D.
I371A	N.D.	N.D.	N.D.	N.D.	3 ± 1^*^ (3)	N.D.
Y372A	6.09 ± 0.09^*^ (4)	9.40 ± 0.06 (4)	7.55 ± 0.27^*^ (4)	9.85 ± 0.19 (8)	98 ± 13 (4)	21,700 ± 8600 (4)
D373A	5.76 ± 0.22^*^ (6)	9.36 ± 0.09^*^ (4)	7.76 ± 0.28 (4)	9.28 ± 0.15 (8)	98 ± 14 (4)	43,500 ± 22,700 (5)
Y374A	6.64 ± 0.17 (4)	9.79 ± 0.13 (4)	8.23 ± 0.09 (4)	9.83 ± 0.13 (8)	65 ± 5^*^ (4)	26,300 ± 7500 (4)
V375A	6.48 ± 0.19 (5)	9.83 ± 0.08 (4)	8.29 ± 0.25 (4)	9.72 ± 0.09 (8)	115 ± 13 (3)	22,500 ± 8000 (4)
M376A	6.06 ± 0.21^*^ (6)	9.80 ± 0.15 (6)	8.11 ± 0.24 (4)	9.88 ± 0.10 (8)	113 ± 7 (4)	23,600 ± 3200 (4)

pK_i_ values were derived for each ligand and mutant receptor from analysis of either homologous (sCT(8-32)) or heterologous (sCT, hCT, pCT) competition of [125]I-sCT(8-32) binding. The number of receptors per cell (Bmax) was estimated from homologous competition studies. All values are mean ± S.E.M. (independent “n” values are indicated within parentheses). Cell surface receptor expression was determined by FACS using an anti-c-myc antibody, and expression normalized to WT expression and expressed as %WT. Significance of changes in receptor expression, or pK_i_ of each ligand, was determined by comparison of mutant receptors to WT values by a one-way analysis of variance and Dunnett’s post-test for affinity data, and two-tailed *t*-test for expression data (p < 0.05 represented by *). (N.D.) affinity not determined as no radioligand binding was detected.

### 3.2 ECL2 and ECL3 mutants differentially modulate peptide-specific affinity

Global affinity was determined by competition of ^125^I-sCT(8-32) binding in whole cells by each of the peptides. Homologous competition with sCT(8-32) revealed a pK_i_ for the WT receptor of 9.70 ± 0.05, and a Bmax of 22,900 ± 2500 sites/cell (Table 1). Overall, there was a good correlation, for the mutant receptors relative to WT, between measured Bmax and cell surface expression data from antibody binding (Fig. 1F), although there was a high error in Bmax estimates, consistent with expectations of cold saturation experiments with only two radioligand concentrations. sCT, hCT and pCT had pKi values for the WT receptor of 9.87 ± 0.03, 6.72 ± 0.06 and 8.27 ± 0.07, respectively (Table 1), consistent with those reported in previous studies [9], [10].

**Fig. 2:**
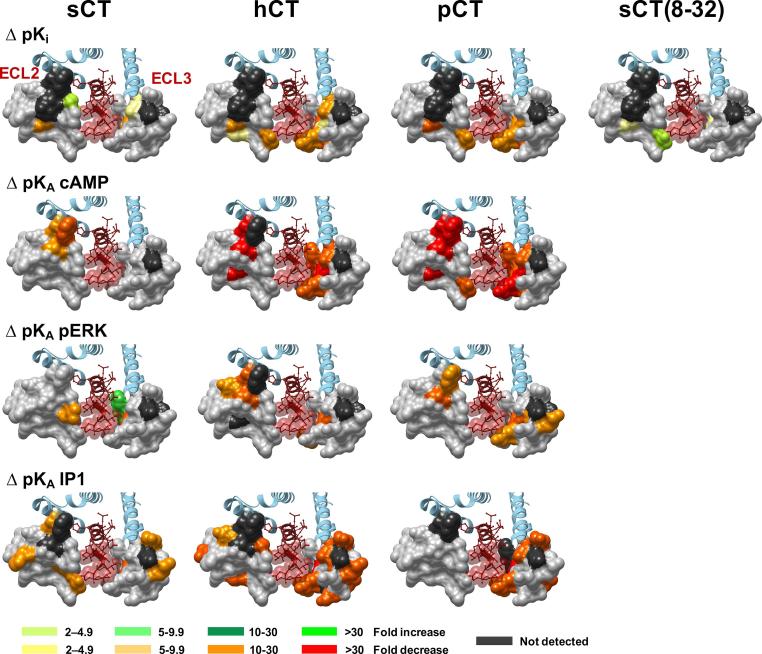
Alanine mutation of ECL2 and ECL3 of the hCTR alters CT peptide binding pK_i_ and functional affinity (pK_A_) in a peptide- and pathway-specific manner. The effect of mutation on peptide affinity in competition for the antagonist radioligand ^125^I-sCT(8-32) is displayed as ΔpK_i_ in the upper panels, with functional affinities derived from operation fitting of concentration-response curves in cAMP accumulation, pERK and IP1 accumulation displayed as ΔpK_A_ in the mid and lower panels, respectively. Illustrated is a top view of the active, sCT-bound, hCTR model with ECL2 and ECL3 shown in surface representation. Mutations that significantly alter peptide affinity are coloured according the magnitude of effect (from Table 1, Table 5), with mutated amino acids without significant alteration to affinity coloured grey. sCT is shown as dark red, with side chains in proximity to the ECLs displayed in x-stick, and residues 1–7 that are critical for receptor activation displayed in transparent cpk. (For interpretation of the references to colour in this figure legend, the reader is referred to the web version of this article.)

**Fig. 3:**
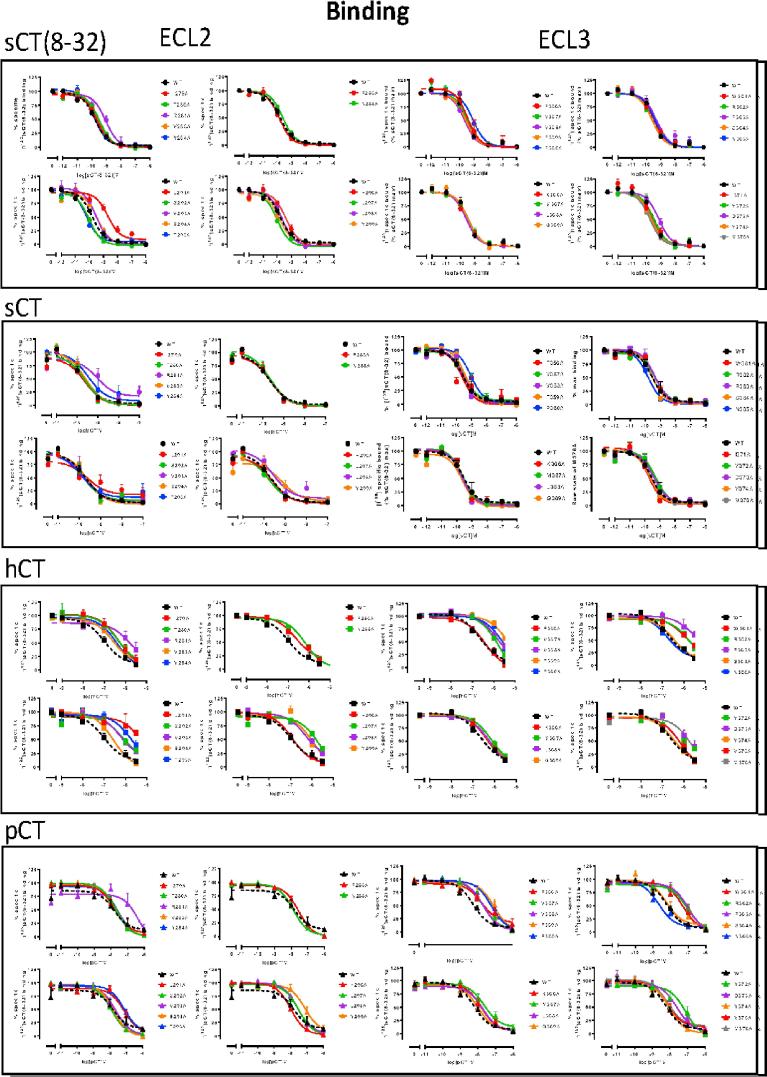
Competition of ^125^I-sCT(8-32) binding by CT peptides for wild-type (WT) and each of the hCTR mutants stably expressed in CV1- FlpIn cells. Whole cell radioligand binding was performed for each receptor mutant in presence of ^125^I-sCT(8-32) and competing peptide ranging in concentration between 1 μM and 1 pM. Non-specific binding was determined in the presence of 1 μM of sCT(8-32) and was used to calculate% of specific binding. Data were fit with a three-parameter logistic equation. All values are mean + S.E.M. of 3–12 (WT 16–18) independent experiments, conducted in duplicate.

Mutants with very low expression, N286A, D287A, C289A, W290A, I300A, K370A and I371A, displayed little or no detectable ^125^I-sCT(8-32) binding, thereby preventing assessment of global affinity, whereas all other mutant receptors exhibited a sufficient radioligand binding window to determine peptide affinity. Of these, only R281A, L291A and P360A reduced sCT(8-32) affinity, whereas T295A increased affinity of this peptide (Table 1, Fig. 2; Fig. 3). Similarly, there was only limited effect on sCT affinity, with reduced affinity for the R281A, P360A and D373A mutants, and increased affinity for S292A. Intriguingly, the L291A mutant that had reduced affinity for the antagonist and both hCT and pCT, did not alter sCT affinity. Both hCT and pCT were more broadly sensitive to mutation, with those affecting pCT common with hCT, including R281A, L291A, T295A, L298A, F359A-P363A and Y372A that principally resided either within 5 Å of sCT in the CTR model, or were involved in the network of amino acids in the core of ECL2 that was linked to receptor stability/expression (Table 1, Fig. 1, Fig. 2). There was a significant reduction in pCT affinity for the Y299A mutant that also trended lower for hCT. Moreover, there was selective, significant attenuation of hCT affinity for L297A, L298A, V357A, V358A, D373A and M376A (Table 1, Fig. 2).

CGRP and Amy displayed no detectable competition with the radioligand at the wildtype or mutant receptors within the concentration range assessed (up to 10 μM), confirming previous findings that these have low affinity for the CTR.

### 3.3 ECL2 and ECL3 mutants alter functional affinity in a ligand and pathway dependent manner

**Fig. 4:**
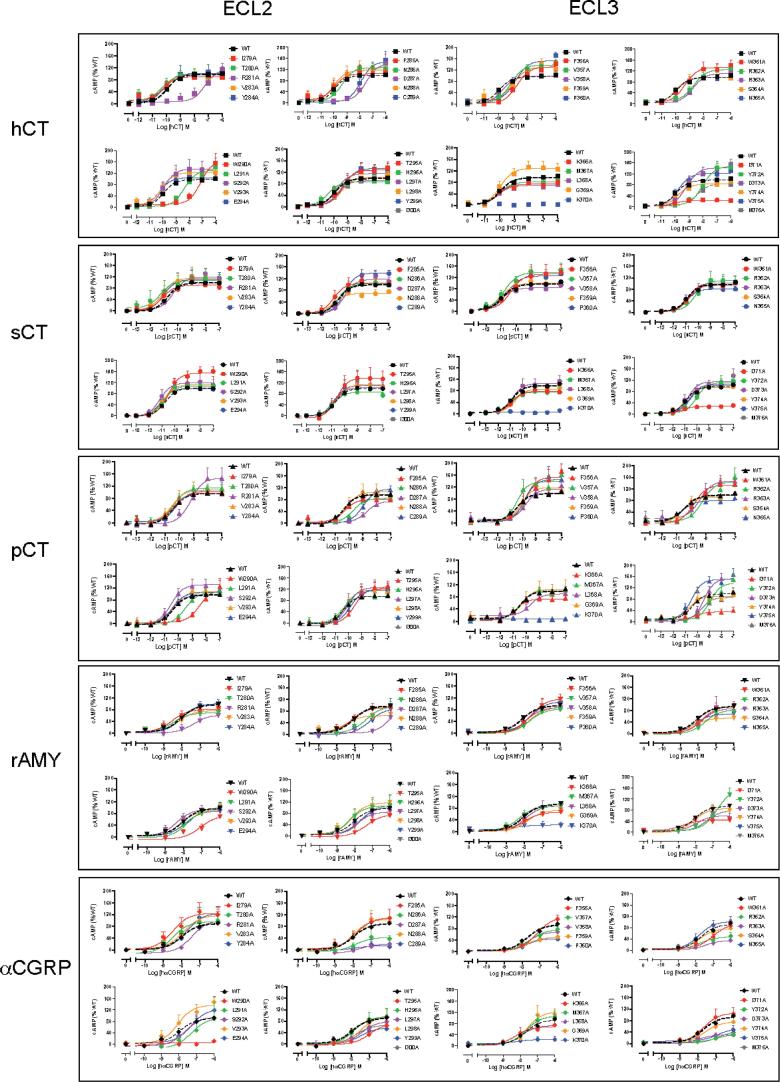
cAMP accumulation profiles elicited by sCT, hCT, pCT, rat amylin (rAmy) or hCGRP in CV1-FlpIn cells stably expressing wild-type (WT) or single alanine mutations of ECL2 or ECL3. cAMP formation in the presence of agonist peptides was normalized to responses of the internal control (0.1 mM forskolin) and fit to a three-parameter logistic equation. Data was subsequently normalized to WT receptor response. All values are mean + S.E.M. of 4–11 (WT 25–36) independent experiments conducted in duplicate.

**Fig. 5:**
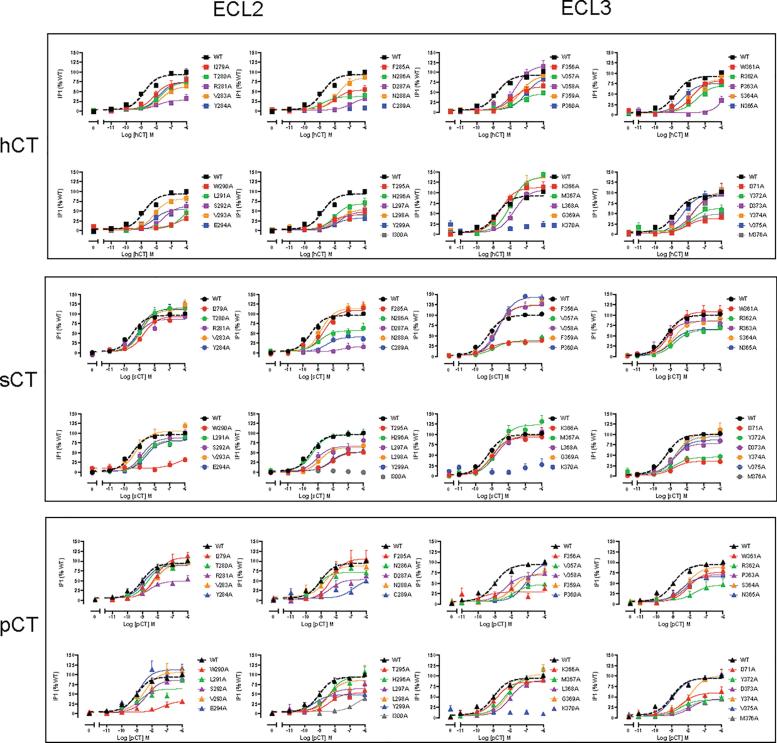
IP1 accumulation profiles elicited by sCT, hCT, or pCT in CV1-FlpIn cells stably expressing wild-type (WT) or single alanine mutations of ECL2 or ECL3. IP1 accumulation in the presence of agonist peptides was normalized to responses of the internal control (0.1 mM ATP) and fit to a three-parameter logistic equation. Data was subsequently normalized to WT receptor response. All values are mean + S.E.M. of 4–7 (WT 37–47) independent experiments conducted in duplicate.

**Fig. 6:**
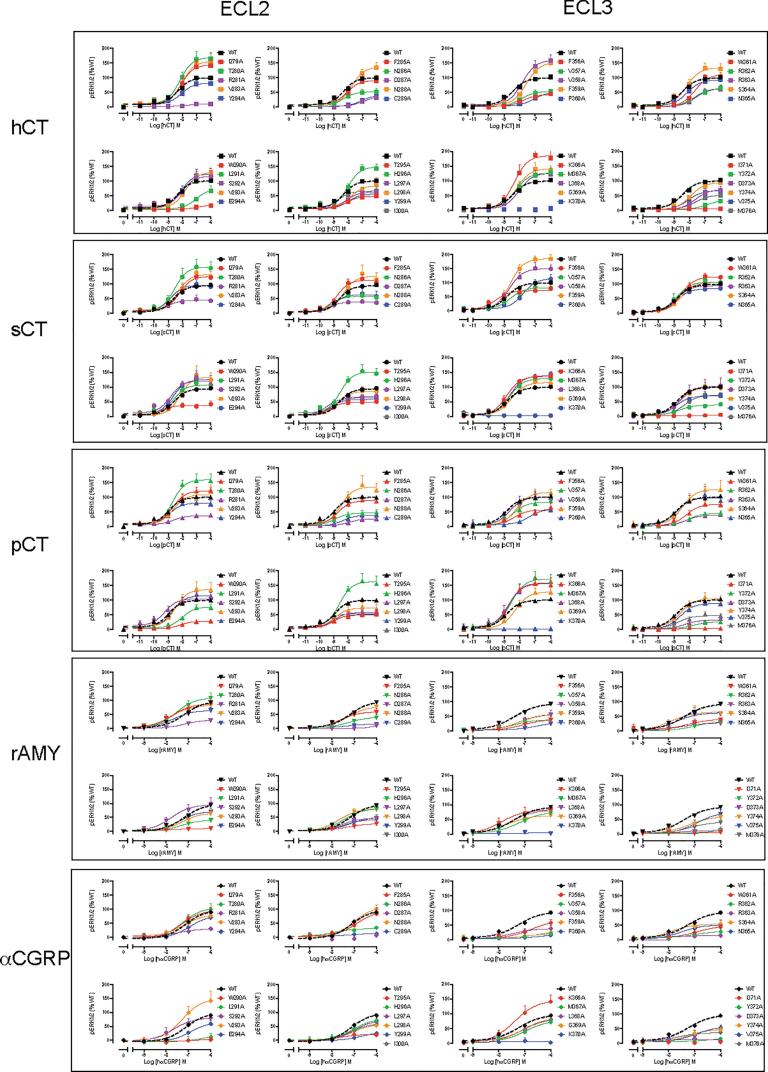
Peak ERK1/2 phosphorylation (pERK) profiles elicited by sCT, hCT, pCT, rat amylin (rAmy) or hCGRP in CV1-FlpIn cells stably expressing wild-type (WT) or single alanine mutations of ECL2 or ECL3. pERK in the presence of agonist peptides was normalized to the WT receptor response and fit to a three-parameter logistic equation. All values are mean + S.E.M. of 4–6 (WT 30–35) independent experiments conducted in duplicate.

**Fig. 7:**
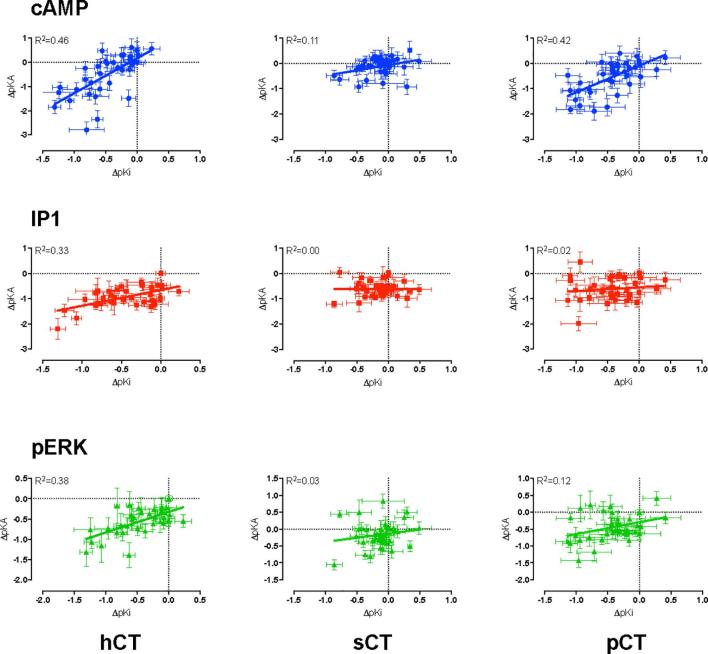
Correlation between changes in global peptide affinity (pK_i_) derived from heterologous competition binding assays, and functional pK_A_ for cAMP (upper panels), IP1 (middle panels) or pERK (bottom panels) signalling, for hCT (left hand panels), sCT (middle panels) or pCT (right hand panels). For all peptides, the highest correlation was seen between pK_i_ and pK_A_ derived from operational analysis of cAMP response data. Significant but weaker correlations were also observed between pK_i_ and functional affinities for IP1 and pERK signalling for hCT, and for pERK signalling alone for pCT. No correlation was observed for sCT pK_A_ values from IP1 or pERK assays or pCT IP1 assays.

**Fig. 8:**
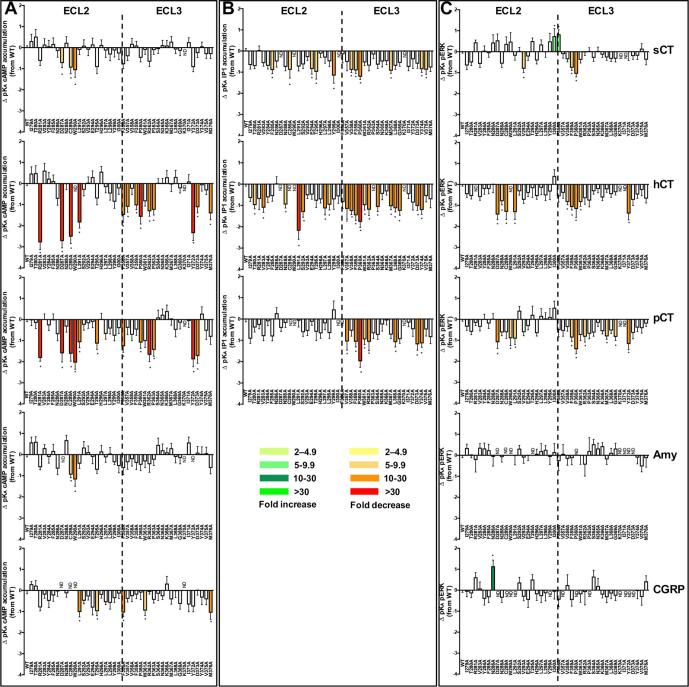
Alanine mutation of ECL2 and ECL3 of the hCTR selectively alters peptide functional affinity in a pathway-dependent manner. For each receptor mutant and ligand, concentration-response data for (A) cAMP accumulation, (B) IP1 accumulation, or (C) pERK assays were fit with the Black and Leff operational model to derive a functional affinity (pK_A_) for each pathway, and these values were subtracted from the WT pK_A_ values to yield ΔpK_A_. All values are mean + S.E.M. of 4–11 (WT 25–47) independent experiments conducted in duplicate. Significance of changes were determined by comparison of the WT to the other receptor mutants in a one-way analysis of variance and Dunnett’s post-test of determined pK_A_ values (Table 5) with significant changes (P < 0.05) denoted by *, and coloured according to the magnitude of effect. N.D., functional affinity not determined.

Concentration-response data, for cAMP accumulation, in response to each peptide was established for WT and mutant receptors (Fig. 4, Table 2), IP1 accumulation (Fig. 5; Table 3), and ERK1/2 phosphorylation (Fig. 6; Table 4). Functional affinity for each of the pathways was determined by operational fitting of the concentration response data. The effect of mutation on pK_A_ for cAMP formation was broadly similar to the derived pK_i_ values (Fig. 6, Fig. 7), clustering to either residues in proximity to the peptide or in the central segment of ECL2 that was important for receptor stability and expression (Fig. 2, Fig. 8). Unlike the competition binding assay, estimates of pK_A_ could be derived for at least one of the peptides for all mutants, except K370A that was not expressed at the cell surface (Fig. 2, Fig. 8, Table 5). Of these, N286A had no impact on affinity, while D287A, C289A and W290A caused a marked decrease in pK_A_ for cAMP formation for all peptides, and these were the only mutations to alter sCT cAMP pK_A_ values. The weak IP1 responses for these mutants made interpretation of effect difficult, however they had clear differential impact on pERK pK_A_ values. While none of the mutants altered sCT pERK pK_A_, D287A decreased both hCT and pCT functional affinities, and W290A decreased that for pCT (hCT was not detectable), and there was a selective loss of pERK functional affinity for hCT at the C289A mutant (Fig. 2, Fig. 8, Table 5).

**Table 2:** Effect of single alanine mutation in hCTR ECL2 or ECL3 on cAMP signalling in response to CT-family peptides.

	hCT			sCT			pCT			rAmy			hαCGRP		
	pEC_50_	E_max_ (% WT)	N	pEC_50_	E_max_ (% WT)	N	pEC_50_	E_max_ (% WT)	N	pEC_50_	E_max_ (% WT)	N	pEC_50_	E_max_ (% WT)	N
WT	9.93 ± 0.06	100 ± 2	(36)	10.7 ± 0.05	100 ± 1	(36)	10.4 ± 0.10	100 ± 2	(31)	8.05 ± 0.11	100 ± 4	(31)	7.96 ± 0.21	100 ± 7	(25)
I279A	10.3 ± 0.18	91 ± 4	(4)	11.1 ± 0.25	90 ± 6	(5)	10.4 ± 0.15	97 ± 4	(6)	8.53 ± 0.35	81 ± 9	(5)	8.47 ± 0.41	139 ± 18	(5)
T280A	10.3 ± 0.28	97 ± 6	(4)	11.3 ± 0.56	107 ± 14	(4)	10.4 ± 0.17	113 ± 5	(5)	8.47 ± 0.38	69 ± 8	(4)	8.48 ± 0.49	107 ± 21	(5)
R281A	7.22 ± 0.26^*^	117 ± 15	(8)	10.4 ± 0.17	119 ± 5	(11)	9.19 ± 0.28^*^	147 ± 16^*^	(6)	7.36 ± 0.5	63 ± 14	(5)	7.41 ± 0.43	114 ± 25	(5)
V283A	10.4 ± 0.34	90 ± 7	(4)	11.1 ± 0.38	116 ± 12	(5)	10.5 ± 0.32	107 ± 9	(5)	8.17 ± 0.36	77 ± 10	(5)	7.89 ± 0.39	130 ± 19	(5)
Y284A	10.2 ± 0.35	103 ± 10	(5)	11.0 ± 0.35	111 ± 11	(5)	10.2 ± 0.29	104 ± 9	(6)	7.99 ± 0.25	101 ± 10	(5)	7.72 ± 0.39	139 ± 22	(5)
F285A	9.97 ± 0.42	105 ± 11	(5)	11.1 ± 0.29	105 ± 8	(5)	10.3 ± 0.45	81 ± 10	(6)	8.17 ± 0.28	92 ± 9	(5)	7.80 ± 0.39	122 ± 18	(5)
N286A	9.37 ± 0.17	126 ± 6	(5)	10.8 ± 0.22	103 ± 6	(5)	9.70 ± 0.22	84 ± 6	(5)	7.35 ± 0.35	97 ± 15	(5)	7.85 ± 0.75	46 ± 16	(5)
D287A	7.53 ± 0.30^*^	140 ± 18^*^	(6)	10.3 ± 0.22	119 ± 7	(10)	8.72 ± 0.34^*^	74 ± 9	(6)	N.D.	N.D.		N.D.	N.D.	
N288A	9.99 ± 0.32	117.0 ± 10	(5)	11.2 ± 0.28	69 ± 5^*^	(6)	10.2 ± 0.31	105 ± 10	(5)	8.52 ± 0.44	64 ± 8	(5)	7.99 ± 0.34	118 ± 17	(5)
C289A	7.92 ± 0.21^*^	148 ± 7^*^	(8)	10.3 ± 0.17	140 ± 7^*^	(10)	8.99 ± 0.16^*^	115 ± 7	(8)	7.17 ± 0.48	89 ± 23	(5)	N.D.	N.D.	
W290A	7.13 ± 0.24^*^	164 ± 20^*^	(10)	10.5 ± 0.16	154 ± 7^*^	(11)	8.48 ± 0.28^*^	113 ± 12	(5)	7.09 ± 0.55	72 ± 26	(5)	N.D.	N.D.	
L291A	8.36 ± 0.11^*^	131 ± 5^*^	(10)	10.9 ± 0.14	108 ± 4	(10)	9.50 ± 0.14^*^	105 ± 5	(6)	7.74 ± 0.31	101 ± 13	(5)	7.29 ± 0.64	101 ± 36	(5)
S292A	9.95 ± 0.35	135 ± 13	(6)	11.1 ± 0.38	119 ± 12	(6)	10.5 ± 0.26	131 ± 9^*^	(6)	8.39 ± 0.32	95 ± 10	(6)	7.65 ± 0.30	106 ± 14	(5)
V293A	10.1 ± 0.24	123 ± 8	(5)	10.9 ± 0.35	112 ± 11	(5)	10.3 ± 0.18	107 ± 5	(6)	8.13 ± 0.29	91 ± 10	(6)	8.06 ± 0.29	153 ± 17	(5)
E294A	10.3 ± 0.20	105 ± 5	(5)	11.1 ± 0.31	98 ± 9	(5)	10.4 ± 0.22	105 ± 6	(5)	7.92 ± 0.26	90 ± 9	(5)	7.31 ± 0.49	137 ± 31	(5)
T295A	9.44 ± 0.31	127 ± 11	(5)	10.5 ± 0.31	138 ± 13^*^	(5)	9.55 ± 0.21	123 ± 8	(5)	7.36 ± 0.48	74 ± 19	(5)	6.97 ± 0.42	79 ± 20	(5)
H296A	10.5 ± 0.19	87 ± 4	(10)	11.0 ± 0.18	86 ± 5	(10)	10.5 ± 0.29	96 ± 8	(5)	8.29 ± 0.48	107 ± 18	(5)	7.85 ± 0.41	106 ± 18	(6)
L297A	9.75 ± 0.25	91 ± 6	(4)	10.8 ± 0.4	103 ± 12	(4)	10.0 ± 0.20	127 ± 8	(6)	7.87 ± 0.30	85 ± 10	(5)	7.44 ± 0.34	88 ± 14	(5)
L298A	9.44 ± 0.20	103 ± 6	(5)	10.7 ± 0.3	112 ± 9	(5)	10.2 ± 0.20	118 ± 7	(5)	8.21 ± 0.30	117 ± 13	(5)	7.47 ± 0.36	109 ± 17	(5)
Y299A	9.33 ± 0.22	133 ± 9	(5)	10.9 ± 0.25	110 ± 7	(5)	9.87 ± 0.23	117 ± 9	(6)	7.71 ± 0.25	102 ± 1	(4)	7.48 ± 0.55	62 ± 16	(5)
I300A	9.83 ± 0.18	116 ± 6	(5)	10.7 ± 0.36	113 ± 11	(5)	10.3 ± 0.30	117 ± 11	(5)	7.61 ± 0.22	109 ± 10	(5)	7.71 ± 0.45	73 ± 14	(5)
F356A	8.71 ± 0.18^*^	131 ± 8^*^	(5)	10.9 ± 0.05	100 ± 1	(6)	10.5 ± 0.07	100 ± 2	(4)	7.62 ± 0.30	126 ± 15	(4)	7.18 ± 0.11	128 ± 10	(5)
V357A	9.15 ± 0.14^*^	137 ± 6^*^	(6)	10.3 ± 0.32	133 ± 12^*^	(6)	9.82 ± 0.35	154 ± 17^*^	(4)	7.69 ± 0.27	88 ± 10	(5)	7.53 ± 0.32	82 ± 10	(5)
V358A	9.72 ± 0.16	98 ± 4	(4)	10.8 ± 0.24	139 ± 9^*^	(4)	10.5 ± 0.38	140 ± 15^*^	(4)	7.86 ± 0.36	94 ± 13	(4)	7.68 ± 0.31	74 ± 9	(5)
F359A	8.95 ± 0.24^*^	130 ± 9^*^	(5)	11.0 ± 0.18	84 ± 4.0	(5)	9.79 ± 0.25	113 ± 8	(4)	7.63 ± 0.25	95 ± 9	(5)	7.73 ± 0.41	53 ± 8^*^	(5)
P360A	8.80 ± 0.17^*^	152 ± 8^*^	(5)	11.0 ± 0.18	100 ± 5	(5)	9.88 ± 0.27	146 ± 13	(4)	7.63 ± 0.30	115 ± 13	(5)	7.76 ± 0.28	48 ± 5^*^	(5)
W361A	9.32 ± 0.25	132 ± 9^*^	(5)	10.6 ± 0.19	126 ± 7^*^	(5)	9.83 ± 0.20	133 ± 9^*^	(4)	7.79 ± 0.31	102 ± 13	(5)	7.01 ± 0.30	102 ± 16	(5)
R362A	8.68 ± 0.24^*^	125 ± 9^*^	(4)	10.8 ± 0.14	95 ± 4	(4)	9.74 ± 0.21	138 ± 9^*^	(4)	7.61 ± 0.32	88 ± 12	(4)	7.75 ± 0.48	51 ± 9^*^	(5)
P363A	8.74 ± 0.13^*^	111 ± 5	(5)	10.3 ± 0.28	111 ± 9	(5)	9.41 ± 0.34^*^	148 ± 15^*^	(4)	7.82 ± 0.55	94 ± 20	(6)	7.41 ± 0.47	38 ± 7^*^	(5)
S364A	9.94 ± 0.36	97 ± 9	(5)	10.8 ± 0.26	102 ± 7	(5)	9.22 ± 0.29^*^	166 ± 16^*^	(4)	8.35 ± 0.38	56 ± 7^*^	(4)	7.78 ± 0.31	83 ± 10	(4)
N365A	9.91 ± 0.16	96 ± 4	(5)	10.8 ± 0.19	94 ± 5	(5)	10.5 ± 0.21	95 ± 5	(4)	8.18 ± 0.32	72 ± 8	(4)	7.81 ± 0.31	111 ± 13	(5)
K366A	10.1 ± 0.17	74 ± 3^*^	(5)	10.9 ± 0.23	81 ± 5^*^	(5)	10.7 ± 0.39	80 ± 8	(4)	8.02 ± 0.26	71 ± 7	(4)	8.27 ± 0.54	73 ± 13	(5)
M367A	10.0 ± 0.19	82 ± 4	(5)	10.9 ± 0.16	79 ± 3^*^	(5)	10.8 ± 0.34	73 ± 13	(4)	8.30 ± 0.26	93 ± 8	(4)	7.67 ± 0.31	114 ± 14	(5)
L368A	10.1 ± 0.23	70 ± 4^*^	(5)	11.1 ± 0.33	75 ± 7^*^	(5)	10.5 ± 0.26	96 ± 6	(4)	7.93 ± 0.34	95 ± 12	(5)	7.94 ± 0.4	78 ± 12	(5)
G369A	9.92 ± 0.27	129 ± 9^*^	(5)	10.8 ± 0.27	106 ± 8	(5)	9.69 ± 0.28	89 ± 7	(4)	7.83 ± 0.20	78 ± 6	(4)	7.61 ± 0.38	131 ± 20	(5)
K370A	N.D.	N.D.		N.D.	N.D.		N.D.	N.D.		N.D.	N.D.		N.D.	N.D.	
I371A	9.99 ± 0.47	24 ± 2.7^*^	(5)	11.2 ± 0.30	28 ± 2^*^	(5)	10.1 ± 0.21	35 ± 5^*^	(4)	8.73 ± 0.60	47 ± 7^*^	(5)	6.88 ± 0.54	58 ± 15^*^	(5)
Y372A	8.03 ± 0.20^*^	149 ± 12.^*^	(4)	10.0 ± 0.27	113 ± 10	(4)	8.94 ± 0.24^*^	137 ± 13^*^	(4)	6.73 ± 0.26	170 ± 26^*^	(5)	6.91 ± 0.45	36 ± 8^*^	(5)
D373A	9.20 ± 0.19	142 ± 8^*^	(4)	10.8 ± 0.25	116 ± 8	(4)	9.41 ± 0.16^*^	158 ± 9^*^	(4)	7.98 ± 0.70	64 ± 16	(5)	7.95 ± 0.41	34 ± 5^*^	(5)
Y374A	9.78 ± 0.23	83.2 ± 5	(5)	10.9 ± 0.18	96 ± 5	(5)	10.7 ± 0.39	90 ± 9	(4)	8.10 ± 0.24	92 ± 9	(4)	7.60 ± 0.36	82 ± 12	(5)
V375A	9.79 ± 0.22	122 ± 7^*^	(5)	10.6 ± 0.17	106 ± 5	(5)	10.5 ± 0.21	151 ± 8^*^	(4)	8.10 ± 0.27	92 ± 9	(5)	7.69 ± 0.38	114 ± 17	(5)
M376A	8.44 ± 0.37^*^	84.3 ± 10	(5)	10.6 ± 0.20	102 ± 6	(5)	9.63 ± 0.40	87 ± 11^*^	(4)	7.33 ± 0.34	85 ± 13	(5)	6.85 ± 0.26	47 ± 7^*^	(5)

For each receptor and ligand, data were fit to a three-parameter logistic equation to derive pEC_50_ (negative logarithm of the concentration of ligand that produces half the maximal response) and Emax (maximal response, as % of WT). All values are mean ± S.E.M. *(independent “n” values are indicated within parentheses)*. For each ligand, significance of changes in pEC50 and Emax were determined by comparison of mutants to the WT receptor in a one-way analysis of variance followed by Dunnett’s post-test (p < 0.05 represented by *). (N.D.) pEC_50_ or Emax not determined.

**Table 3:** Effect of single alanine mutation in hCTR ECL2 or ECL3 on IP1 signalling in response to CT peptides.

	hCT			sCT			pCT		
	pEC_50_	E_max_ (% WT)	N	pEC_50_	E_max_ (% WT)	N	pEC_50_	E_max_ (% WT)	N
WT	8.80 ± 0.07	100 ± 3	(47)	9.24 ± 0.5	100 ± 3	(44)	8.86 ± 0.13	100 ± 3	(37)
I279A	8.08 ± 0.25	79 ± 7	(7)	8.72 ± 0.23	97 ± 7	(7)	8.18 ± 0.22	116 ± 10	(5)
T280A	7.68 ± 0.20^*^	79 ± 6	(6)	8.89 ± 0.12	119 ± 5	(7)	8.61 ± 0.21	100 ± 6	(7)
R281A	7.96 ± 0.37^*^	30 ± 4	(7)	9.03 ± 0.23	91 ± 6	(6)	8.65 ± 0.23	52 ± 4^*^	(6)
V283A	7.60 ± 0.21	67 ± 6^*^	(5)	8.87 ± 0.15	115 ± 6	(7)	8.21 ± 0.14	100 ± 5	(7)
Y284A	7.71 ± 0.37	81 ± 12	(7)	8.94 ± 0.20	118 ± 7	(6)	8.94 ± 0.20	94 ± 6	(7)
F285A	7.99 ± 0.27	58 ± 6^*^	(6)	8.59 ± 0.17	114 ± 6	(5)	8.30 ± 0.32	112 ± 13	(6)
N286A	8.82 ± 0.28	40 ± 3^*^	(6)	8.93 ± 0.26	59 ± 5^*^	(5)	9.25 ± 0.27	76 ± 6	(7)
D287A	N.D	N.D		N.D.	N.D.		8.43 ± 0.38	56 ± 7^*^	(6)
N288A	7.78 ± 0.22^*^	91 ± 8	(7)	8.80 ± 0.15	119 ± 6	(6)	8.72 ± 0.25	93 ± 7	(7)
C289A	N.D.	N.D.		8.52 ± 0.28	42 ± 4^*^	(6)	N.D	N.D	(7)
W290A	N.D.	N.D.		N.D	N.D		N.D	N.D	
L291A	6.28 ± 0.53^*^	71 ± 16^*^	(7)	8.73 ± 0.14	85 ± 4	(7)	8.79 ± 0.31	67 ± 6^*^	(7)
S292A	7.51 ± 0.20^*^	70 ± 6	(6)	8.86 ± 0.21	91 ± 6	(7)	8.37 ± 0.23	92 ± 7	(7)
V293A	8.12 ± 0.13	87 ± 4	(6)	9.21 ± 0.22	109 ± 7	(7)	8.49 ± 0.27	112 ± 10	(5)
E294A	8.21 ± 0.27	57 ± 6^*^	(6)	8.62 ± 0.18	84 ± 5	(6)	8.89 ± 0.22	119 ± 8	(6)
T295A	8.31 ± 0.27	43 ± 4^*^	(6)	8.11 ± 0.33^*^	53 ± 7^*^	(6)	8.34 ± 0.37	56 ± 7^*^	(6)
H296A	7.98 ± 0.19^*^	73 ± 6^*^	(7)	9.34 ± 0.19	101 ± 5	(7)	8.35 ± 0.21	101 ± 7	(6)
L297A	7.68 ± 0.25^*^	52 ± 5^*^	(7)	9.12 ± 0.32	69 ± 7^*^	(6)	8.70 ± 0.25	68 ± 5^*^	(7)
L298A	7.69 ± 1.10	58 ± 6^*^	(7)	8.88 ± 0.22	66 ± 5^*^	(7)	8.39 ± 0.25	91 ± 10	(5)
Y299A	7.94 ± 0.37	34 ± 5^*^	(6)	8.19 ± 0.27^*^	54 ± 5^*^	(6)	9.38 ± 0.25	52 ± 3^*^	(7)
I300A	8.12 ± 0.37	44 ± 4^*^	(6)	N.D	N.D		N.D.	N.D.	
F356A	7.95 ± 0.20^*^	69 ± 6^*^	(6)	9.23 ± 0.29	36 ± 3^*^	(6)	N.D	N.D	
V357A	7.61 ± 0.23^*^	50 ± 4^*^	(5)	8.90 ± 0.20	39 ± 3^*^	(5)	7.81 ± 0.20^*^	48 ± 4^*^	(5)
V358A	7.64 ± 0.18^*^	126 ± 9^*^	(6)	8.76 ± 0.15	123 ± 6^*^	(6)	8.48 ± 0.26	73 ± 6^*^	(5)
F359A	7.29 ± 0.20^*^	101 ± 9	(5)	8.72 ± 0.14	125 ± 6^*^	(5)	7.88 ± 0.18^*^	77 ± 6	(5)
P360A	7.06 ± 0.16^*^	95 ± 8	(5)	8.55 ± 0.11^*^	144 ± 5^*^	(6)	7.07 ± 0.23^*^	107 ± 12	(5)
W361A	7.77 ± 0.26	89 ± 9	(6)	9.01 ± 0.18	108 ± 6	(5)	8.08 ± 0.19^*^	80 ± 6	(6)
R362A	7.47 ± 0.34^*^	77 ± 11	(6)	8.74 ± 0.22	64 ± 5^*^	(5)	7.71 ± 0.50^*^	47 ± 9^*^	(6)
P363A	N.D.	N.D.		9.27 ± 0.35	86 ± 9	(5)	8.21 ± 0.38	73 ± 10^*^	(6)
S364A	7.77 ± 0.21^*^	93 ± 8	(5)	8.79 ± 0.21	87 ± 6	(5)	8.16 ± 0.29	93 ± 6	(5)
N365A	8.36 ± 0.21	81 ± 6	(5)	8.96 ± 0.18	66 ± 4^*^	(4)	8.65 ± 0.29	68 ± 6^*^	(5)
K366A	8.58 ± 0.16	121 ± 6	(5)	9.03 ± 0.15	93 ± 5	(5)	8.83 ± 0.23	91 ± 6	(5)
M367A	7.94 ± 0.14	149 ± 8^*^	(4)	8.71 ± 0.16	123 ± 6^*^	(6)	8.29 ± 0.23	101 ± 8	(5)
L368A	7.76 ± 0.14^*^	115 ± 7	(6)	8.79 ± 0.14	97 ± 4	(5)	8.00 ± 0.24^*^	94 ± 9	(5)
G369A	7.97 ± 0.17^*^	148 ± 10^*^	(5)	8.93 ± 0.22	97 ± 7	(5)	8.12 ± 0.14	110 ± 6	(5)
K370A	N.D.	N.D.		N.D.	N.D.		N.D.	N.D.	
I371A	8.09 ± 0.20	42 ± 3^*^	(7)	8.83 ± 0.21	31 ± 14^*^	(6)	8.38 ± 0.29	61 ± 6^*^	(5)
Y372A	7.83 ± 0.36	68 ± 9^*^	(5)	8.74 ± 0.29	46 ± 4^*^	(7)	8.54 ± 0.35	44 ± 5^*^	(5)
D373A	7.75 ± 0.26^*^	102 ± 10	(5)	8.79 ± 0.26	78 ± 6	(5)	7.83 ± 0.18^*^	46 ± 3^*^	(5)
Y374A	7.65 ± 0.20^*^	110 ± 9	(5)	8.67 ± 0.19^*^	102 ± 6	(6)	7.91 ± 0.15^*^	106 ± 6	(5)
V375A	8.34 ± 0.17	104 ± 6	(5)	8.72 ± 0.21	87 ± 5	(6)	8.93 ± 0.16	100 ± 5	(5)
M376A	8.08 ± 0.26	52 ± 5^*^	(4)	8.67 ± 0.13	95 ± 4	(4)	8.25 ± 0.27	47 ± 4^*^	(5)

For each receptor and ligand, data were fit to a three-parameter logistic equation to derive pEC_50_ (negative logarithm of the concentration of ligand that produces half the maximal response) and Emax (maximal response, as % of WT). All values are mean ± S.E.M. (independent “n” values are indicated within parentheses). For each ligand, significance of changes in pEC50 and Emax were determined by comparison of mutants to the WT receptor in a one-way analysis of variance followed by Dunnett’s post-test (p < 0.05 represented by *). (N.D.) pEC_50_ or Emax not determined.

**Table 4:** Effect of single alanine mutation in hCTR ECL2 or ECL3 on pERK signalling in response to CT-family peptides.

	hCT			sCT			pCT			rAmy			hαCGRP		
	pEC_50_	E_max_ (% WT)	N	pEC_50_	E_max_ (% WT)	N	pEC_50_	E_max_ (% WT)	N	pEC_50_	E_max_ (% WT)	N	pEC_50_	E_max_ (% WT)	N
WT	8.32 ± 0.09	100.0 ± 3	(34)	8.62 ± 0.10	100 ± 3	(35)	8.95 ± 0.08	100 ± 3	(32)	7.19 ± 0.10	100 ± 5	(30)	7.11 ± 0.11	100 ± 6	(30)
I279A	8.09 ± 0.19	143 ± 10^*^	(5)	8.22 ± 0.11	126 ± 5^*^	(5)	8.66 ± 0.17	122 ± 7	(5)	7.74 ± 0.27	88 ± 9	(5)	7.30 ± 0.23	97 ± 11	(5)
T280A	8.17 ± 0.19	168 ± 12^*^	(6)	8.55 ± 0.25	158 ± 12^*^	(6)	8.69 ± 0.19	160 ± 10^*^	(6)	7.57 ± 0.23	110 ± 11	(6)	7.27 ± 0.23	106 ± 12	(6)
R281A	N.D.	N.D.		9.01 ± 0.43	45 ± 5^*^	(5)	8.64 ± 0.35	37 ± 4^*^	(5)	7.16 ± 0.27	31 ± 5^*^	(5)	7.46 ± 0.43	30 ± 5^*^	(5)
V283A	8.00 ± 0.24	155 ± 14^*^	(5)	8.30 ± 0.22	133 ± 10^*^	(6)	8.65 ± 0.34	109 ± 12	(5)	7.77 ± 0.27	83 ± 9	(5)	7.22 ± 0.18	81 ± 7	(4)
Y284A	8.08 ± 0.23	82 ± 7	(5)	8.83 ± 0.21	96 ± 6	(5)	8.92 ± 0.25	81 ± 6	(5)	7.69 ± 0.23	64 ± 6^*^	(5)	6.95 ± 0.31	79 ± 15	(5)
F285A	8.09 ± 0.33	91 ± 11	(5)	8.78 ± 0.16	114 ± 6	(5)	8.74 ± 0.20	88 ± 5	(5)	7.66 ± 0.28	60 ± 7^*^	(5)	6.90 ± 0.34	93 ± 18	(5)
N286A	8.37 ± 0.32	52 ± 5^*^	(5)	9.16 ± 0.28	59 ± 5^*^	(5)	8.92 ± 0.36	46 ± 5^*^	(5)	7.27 ± 0.32	41 ± 6^*^	(5)	7.89 ± 0.47	31 ± 5^*^	(5)
D287A	7.01 ± 0.25^*^	35 ± 5^*^	(5)	9.40 ± 0.23	39 ± 2^*^	(5)	7.9 ± 0.25^*^	27 ± 3^*^	(5)	N.D.	N.D.		N.D.	N.D.	
N288A	7.74 ± 0.15	137 ± 8^*^	(5)	8.36 ± 0.29	127 ± 13^*^	(5)	8.53 ± 0.26	135 ± 11^*^	(5)	7.28 ± 0.2	79 ± 7	(5)	7.01 ± 0.16	110 ± 10	(5)
C289A	7.10 ± 0.39^*^	40 ± 8^*^	(5)	9.07 ± 0.57	64 ± 11^*^	(5)	8.37 ± 0.28	38 ± 4^*^	(5)	N.D.	N.D.		N.D.	N.D.	
W290A	N.D.	N.D.		9.47 ± 0.41	38 ± 4^*^	(5)	8.13 ± 0.26	28 ± 3^*^	(5)	N.D.	N.D.		N.D.	N.D.	
L291A	7.05 ± 0.24^*^	72 ± 9	(5)	8.61 ± 0.15	107 ± 5	(5)	8.02 ± 0.18^*^	77 ± 5	(5)	7.35 ± 0.22	41 ± 4^*^	(5)	N.D.	N.D.	
S292A	8.02 ± 0.26	116 ± 11	(5)	8.84 ± 0.21	121 ± 7	(5)	9.26 ± 0.25	104 ± 7	(5)	7.76 ± 0.31	96 ± 12	(6)	7.68 ± 0.35	80 ± 12	(5)
V293A	7.78 ± 0.15	130 ± 8^*^	(5)	8.18 ± 0.24	135 ± 12^*^	(5)	8.50 ± 0.25	136 ± 11^*^	(5)	7.29 ± 0.26	67 ± 8^*^	(5)	7.28 ± 0.31	149 ± 22	(5)
E294A	8.07 ± 0.21	125 ± 10	(5)	8.67 ± 0.17	126 ± 7^*^	(5)	8.70 ± 0.24	115 ± 9	(5)	7.43 ± 0.22	72 ± 7	(5)	6.91 ± 0.36	66 ± 15	(5)
T295A	8.13 ± 0.34	48 ± 6^*^	(5)	9.27 ± 0.30	49 ± 4^*^	(5)	9.12 ± 0.32	50 ± 5^*^	(5)	7.35 ± 0.39	28 ± 5^*^	(5)	7.68 ± 0.38	22 ± 4^*^	(5)
H296A	8.02 ± 0.14	148 ± 8^*^	(5)	8.57 ± 0.20	152 ± 9^*^	(5)	8.63 ± 0.20	163 ± 10^*^	(6)	7.47 ± 0.22	82 ± 8	(5)	7.04 ± 0.27	77 ± 11	(5)
L297A	8.10 ± 0.22	63 ± 5^*^	(5)	9.05 ± 0.22	68 ± 4^*^	(5)	9.01 ± 0.26	59 ± 4^*^	(5)	7.55 ± 0.27	49 ± 6^*^	(5)	7.01 ± 0.29	73 ± 12	(5)
L298A	7.82 ± 0.29	84 ± 10	(5)	8.75 ± 0.21	85 ± 6	(5)	8.96 ± 0.28	73 ± 6^*^	(5)	7.69 ± 0.20	82 ± 7	(5)	7.12 ± 0.24	58 ± 8	(5)
Y299A	7.99 ± 0.28	56 ± 6^*^	(5)	9.23 ± 0.25	58 ± 4^*^	(5)	9.03 ± 0.19	51 ± 3^*^	(5)	7.22 ± 0.28	51 ± 7^*^	(5)	N.D	N.D	
I300A	8.71 ± 0.29	65 ± 6^*^	(5)	9.49 ± 0.21	63 ± 3^*^	(5)	9.55 ± 0.22	54 ± 3^*^	(5)	7.48 ± 0.25	44 ± 5^*^	(5)	7.17 ± 0.42	61 ± 13	(5)
F356A	7.77 ± 0.17^*^	43 ± 3^*^	(4)	9.52 ± 0.25^*^	72 ± 5^*^	(5)	8.35 ± 0.30	57 ± 6^*^	(4)	7.06 ± 0.23	41 ± 5^*^	(5)	6.74 ± 0.28	68 ± 11	(5)
V357A	7.92 ± 0.21	53 ± 5^*^	(4)	8.61 ± 0.27	82 ± 7^*^	(5)	8.43 ± 0.36	83 ± 10	(4)	7.46 ± 0.46	40 ± 8^*^	(5)	N.D.	N.D.	
V358A	7.83 ± 0.15^*^	160 ± 10^*^	(5)	8.68 ± 0.16	150 ± 8^*^	(4)	8.65 ± 0.26	101 ± 8	(4)	7.23 ± 0.35	59 ± 10^*^	(5)	7.49 ± 0.50	39 ± 8^*^	(5)
F359A	7.57 ± 0.12^*^	153 ± 8^*^	(5)	8.62 ± 0.12	186 ± 7^*^	(5)	8.20 ± 0.15	116 ± 6	(5)	7.22 ± 0.4	58 ± 11^*^	(5)	8.20 ± 0.15	116 ± 6	(5)
P360A	7.12 ± 0.24^*^	51 ± 6^*^	(5)	7.78 ± 0.19^*^	115 ± 9	(5)	7.40 ± 0.28^*^	59 ± 7^*^	(5)	N.D.	N.D.		N.D.	N.D.	
W361A	7.64 ± 0.12^*^	112 ± 6	(5)	8.52 ± 0.12	125 ± 5^*^	(4)	8.20 ± 0.22	76 ± 6^*^	(5)	7.24 ± 0.49	42 ± 9^*^	(4)	6.93 ± 0.31	49 ± 8^*^	(5)
R362A	7.68 ± 0.25^*^	64 ± 7^*^	(5)	8.81 ± 0.28	107 ± 10	(4)	8.12 ± 0.20	45 ± 4^*^	(5)	6.94 ± 0.36	36 ± 7^*^	(4)	6.95 ± 0.61	31 ± 8^**^	(5)
P363A	7.59 ± 0.23^*^	64 ± 6^*^	(5)	8.81 ± 0.15	95 ± 5	(4)	8.24 ± 0.15	39 ± 2^*^	(5)	7.51 ± 0.81	28 ± 9^*^	(4)	N.D.	N.D.	
S364A	8.18 ± 0.20	135 ± 10^*^	(5)	8.56 ± 0.13	106 ± 4	(4)	8.44 ± 0.28	127 ± 12^*^	(4)	7.94 ± 0.36	66 ± 9^*^	(4)	7.97 ± 0.29	54 ± 6^*^	(4)
N365A	8.15 ± 0.16	93 ± 6	(5)	8.82 ± 0.12	84 ± 3	(4)	8.79 ± 0.32	98 ± 10	(5)	7.73 ± 0.39	63 ± 10^*^	(5)	7.52 ± 0.39	52 ± 8^*^	(4)
K366A	8.33 ± 0.24	186 ± 16^*^	(5)	8.84 ± 0.11	136 ± 5^*^	(4)	8.72 ± 0.18	156 ± 9^*^	(5)	7.89 ± 0.33	84 ± 11	(5)	7.39 ± 0.27	146 ± 17^*^	(4)
M367A	8.26 ± 0.15	127 ± 7^*^	(5)	8.66 ± 0.10	130 ± 4^*^	(4)	8.56 ± 0.19	172 ± 10^*^	(5)	7.19 ± 0.15	77 ± 5	(5)	6.97 ± 0.27	78 ± 12	(4)
L368A	8.05 ± 0.14	126 ± 7^*^	(4)	8.62 ± 0.12	140 ± 6^*^	(4)	8.71 ± 0.23	159 ± 11^*^	(5)	7.56 ± 0.23	82 ± 8	(4)	7.07 ± 0.37	84 ± 16	(4)
G369A	8.20 ± 0.18	141 ± 9^*^	(5)	8.53 ± 0.11	115 ± 4	(4)	8.28 ± 0.18	128 ± 8^*^	(5)	7.73 ± 0.34	63 ± 8^*^	(4)	7.09 ± 0.30	87 ± 14	(4)
K370A	N.D.	N.D.		N.D.	N.D.		N.D.	N.D.		N.D.	N.D.		N.D.	N.D.	
I371A	N.D.	N.D.		N.D.	N.D.		N.D.	N.D.		N.D.	N.D.		N.D.	N.D.	
Y372A	8.74 ± 0.29	46 ± 4^*^	(5)	8.64 ± 0.26	39 ± 3^*^	(4)	7.72 ± 0.41^*^	26 ± 4^*^	(4)	N.D.	N.D.		N.D.	N.D.	
D373A	7.63 ± 0.24^*^	71 ± 7^*^	(5)	8.62 ± 0.34	103 ± 11	(4)	8.31 ± 0.35	32 ± 4^*^	(5)	N.D.	N.D.		N.D.	N.D.	
Y374A	7.92 ± 0.09	94 ± 4	(5)	8.63 ± 0.14	99 ± 5	(4)	8.57 ± 0.24	105 ± 8	(4)	7.21 ± 0.17	61 ± 5^*^	(4)	7.21 ± 0.31	49 ± 8^*^	(5)
V375A	8.08 ± 0.13	67 ± 3^*^	(4)	8.83 ± 0.14	70 ± 3^*^	(5)	8.58 ± 0.14	89 ± 4	(5)	6.91 ± 0.29	77 ± 13	(5)	6.96 ± 0.24	58 ± 7^*^	(5)
M376A	7.58 ± 0.21^*^	52 ± 4^*^	(4)	8.35 ± 0.2	72 ± 5^*^	(4)	8.71 ± 0.29	47 ± 4^*^	(5)	7.17 ± 0.32	43 ± 7^*^	(5)	7.74 ± 0.60	36 ± 8^*^	(5)

For each receptor and ligand, data were fit to a three-parameter logistic equation to derive pEC_50_ (negative logarithm of the concentration of ligand that produces half the maximal response) and Emax (maximal response, as % of WT). All values are mean ± S.E.M. (independent “n” values are indicated within parentheses). For each ligand, significance of changes in pEC50 and Emax were determined by comparison of mutants to the WT receptor in a one-way analysis of variance followed by Dunnett’s post-test (p < 0.05 represented by *). (N.D.) pEC_50_ or Emax not determined.

**Table 5:** Effect of single alanine mutation in hCTR ECL2 or ECL3 on functional affinities of CT-family peptides.

	hCT			sCT			pCT			rAmy		hαCGRP	
	cAMP	IP1	pERK1/2	cAMP	IP1	pERK1/2	cAMP	IP1	pERK1/2	cAMP	pERK1/2	cAMP	pERK1/2
WT	9.54 ± 0.09 (36)	8.54 ± 0.10 (47)	8.15 ± 0.08 (34)	10.5 ± 0.09 (36)	9.2 ± 0.12 (44)	8.55 ± 0.07 (35)	10.1 ± 0.10 (31)	8.75 ± 0.13 (37)	8.74 ± 0.09 (32)	7.72 ± 0.11 (31)	7.23 ± 0.11 (30)	7.69 ± 0.16 (25)	7.10 ± 0.11 (30)
I279A	10.0 ± 0.32 (4)	7.89 ± 0.16 (7)	7.68 ± 0.16 (5)	10.8 ± 0.32 (5)	8.54 ± 0.19 (7)	7.89 ± 0.19 (5)	10.1 ± 0.26 (6)	7.82 ± 0.22^*^ (5)	8.39 ± 0.18 (5)	8.30 ± 0.25 (5)	7.58 ± 0.19 (5)	7.99 ± 0.12 (5)	7.03 ± 0.24 (5)
T280A	10.0 ± 0.35 (4)	7.56 ± 0.18^*^ (6)	7.59 ± 0.14 (6)	11.0 ± 0.35 (4)	8.49 ± 0.13 (7)	8.04 ± 0.15 (6)	9.96 ± 0.26 (5)	8.35 ± 0.21 (7)	8.15 ± 0.14 (6)	8.32 ± 0.25 (5)	7.27 ± 0.15 (6)	7.92 ± 0.24 (5)	6.95 ± 0.20 (6)
R281A	6.74 ± 0.34^*^ (8)	7.84 ± 0.50 (7)	N.D.	9.89 ± 0.21 (11)	9.24 ± 0.20 (6)	8.97 ± 0.11 (5)	8.30 ± 0.15^*^ (6)	8.47 ± 0.18 (6)	8.57 ± 0.12 (5)	7.12 ± 0.12 (5)	7.01 ± 0.58 (5)	6.89 ± 0.16 (5)	7.71 ± 0.22 (5)
V283A	10.2 ± 0.35 (4)	7.43 ± 0.22^*^ (5)	7.55 ± 0.15 (5)	10.7 ± 0.28 (5)	8.63 ± 0.14 (7)	7.97 ± 0.17 (6)	10.1 ± 0.27 (5)	7.94 ± 0.21 (7)	8.38 ± 0.20 (5)	8.03 ± 0.17 (5)	7.59 ± 0.19 (5)	7.49 ± 0.17 (5)	7.18 ± 0.31 (4)
Y284A	9.77 ± 0.29 (5)	7.92 ± 0.24 (7)	7.93 ± 0.25 (5)	10.6 ± 0.28 (5)	8.51 ± 0.15 (6)	8.60 ± 0.23 (5)	9.85 ± 0.25 (6)	8.64 ± 0.21 (7)	8.73 ± 0.25 (5)	7.61 ± 0.30 (5)	7.55 ± 0.25 (5)	7.20 ± 0.32 (5)	6.69 ± 0.30 (5)
F285A	9.65 ± 0.29 (5)	7.98 ± 0.24 (6)	7.93 ± 0.23 (5)	10.7 ± 0.29 (5)	8.30 ± 0.17^*^ (5)	8.48 ± 0.20 (5)	10.1 ± 0.30 (6)	7.93 ± 0.21 (6)	8.54 ± 0.23 (5)	7.89 ± 0.31 (5)	7.47 ± 0.28 (5)	7.46 ± 0.28 (5)	6.76 ± 0.26 (5)
N286A	8.81 ± 0.30 (5)	8.56 ± 0.32 (6)	8.16 ± 0.41 (5)	10.4 ± 0.30 (5)	8.71 ± 0.27 (6)	8.99 ± 0.37 (5)	9.43 ± 0.32 (5)	9.02 ± 0.26 (7)	8.75 ± 0.26 (5)	7.05 ± 0.27 (5)	7.16 ± 0.44 (5)	7.65 ± 0.23 (5)	8.25 ± 0.29^*^ (5)
D287A	6.81 ± 0.32^*^ (6)	N.D.	6.71 ± 0.24^*^ (5)	9.79 ± 0.22^*^ (10)	N.D.	9.09 ± 0.30 (5)	8.52 ± 0.42^*^ (6)	8.33 ± 0.17 (6)	7.65 ± 0.30^*^ (5)	N.D.	N.D.	N.D.	N.D.
N288A	9.52 ± 0.27 (5)	7.58 ± 0.16^*^ (7)	7.35 ± 0.17^*^ (6)	10.8 ± 0.29 (6)	8.45 ± 0.19 (6)	7.98 ± 0.18 (5)	9.79 ± 0.27 (5)	8.56 ± 0.21 (7)	8.11 ± 0.16 (6)	8.40 ± 0.23 (5)	−7.15 ± 0.24 (5)	7.55 ± 0.18 (5)	6.74 ± 0.22 (5)
C289A	7.02 ± 0.33^*^ (8)	N.D.	6.84 ± 0.36^*^ (5)	9.57 ± 0.3^*^ (10)	8.31 ± 0.39^*^ (6)	8.92 ± 0.34 (5)	8.50 ± 0.35^*^ (8)	N.D.	8.13 ± 0.30 (5)	6.77 ± 0.15^*^ (5)	N.D.	N.D.	N.D.
W290A	N.D.	N.D.	N.D.	9.45 ± 0.34^*^ (11)	N.D.	9.03 ± 0.43 (5)	8.08 ± 0.31^*^ (5)	N.D.	7.84 ± 0.35^*^ (5)	6.55 ± 0.32^*^ (5)	N.D.	N.D.	N.D.
L291A	7.69 ± 0.27^*^ (10)	6.35 ± 0.4^*^ (7)	6.83 ± 0.36^*^ (5)	10.4 ± 0.23 (10)	8.47 ± 0.21 (7)	8.36 ± 0.21 (5)	9.05 ± 0.27^*^ (6)	8.67 ± 0.30 (7)	7.82 ± 0.28^*^ (5)	7.29 ± 0.23 (5)	7.21 ± 0.43 (5)	6.67 ± 0.24^*^ (5)	N.D.
S292A	9.24 ± 0.29 (6)	7.23 ± 0.25^*^ (6)	7.82 ± 0.18 (5)	10.6 ± 0.28 (6)	8.57 ± 0.19 (7)	8.58 ± 0.18 (5)	9.87 ± 0.26 (6)	8.13 ± 0.20 (7)	9.15 ± 0.20 (5)	8.05 ± 0.28 (6)	7.55 ± 0.16 (6)	7.20 ± 0.15 (5)	7.47 ± 0.25 (5)
V293A	9.58 ± 0.28 (5)	8.01 ± 0.15 (7)	7.41 ± 0.18 (5)	10.4 ± 0.28 (5)	8.92 ± 0.16 (7)	7.74 ± 0.18^*^ (5)	9.95 ± 0.25 (6)	8.19 ± 0.22 (7)	8.13 ± 0.17 (5)	7.84 ± 0.29 (6)	7.12 ± 0.28 (5)	7.41 ± 0.17 (5)	6.79 ± 0.19 (5)
E294A	9.84 ± 0.29 (5)	8.05 ± 0.21 (6)	7.74 ± 0.17 (5)	10.7 ± 0.30 (5)	8.36 ± 0.24^*^ (6)	8.32 ± 0.19 (5)	10.0 ± 0.27 (5)	8.62 ± 0.17 (6)	8.42 ± 0.18 (5)	7.61 ± 0.32 (5)	7.24 ± 0.25 (5)	6.88 ± 0.35 (5)	6.64 ± 0.36 (5)
T295A	8.83 ± 0.30 (5)	7.76 ± 0.32 (6)	7.97 ± 0.44 (5)	9.60 ± 0.31 (5)	8.22 ± 0.34^*^ (6)	9.05 ± 0.15 (5)	8.98 ± 0.28^*^ (5)	8.15 ± 0.41 (6)	8.96 ± 0.41 (5)	7.00 ± 0.17 (5)	N.D.	6.71 ± 0.21^*^ (5)	7.61 ± 0.24 (5)
H296A	10.1 ± 0.25 (10)	7.83 ± 0.18 (7)	7.59 ± 0.16 (5)	10.6 ± 0.24 (10)	9.08 ± 0.18 (7)	8.13 ± 0.17 (5)	10.1 ± 0.29 (5)	8.07 ± 0.23 (6)	8.08 ± 0.14 (6)	7.87 ± 0.29 (5)	7.30 ± 0.22 (5)	7.48 ± 0.16 (6)	6.91 ± 0.29 (5)
L297A	9.37 ± 0.27 (4)	7.40 ± 0.31^*^ (7)	7.99 ± 0.33 (5)	10.4 ± 0.33 (4)	8.89 ± 0.27 (6)	8.90 ± 0.22 (5)	9.44 ± 0.25 (6)	8.54 ± 0.30 (7)	8.91 ± 0.35 (5)	7.59 ± 0.24 (5)	7.43 ± 0.35 (5)	7.14 ± 0.12 (5)	6.82 ± 0.31 (5)
L298A	9.07 ± 0.31 (5)	7.58 ± 0.25^*^ (7)	7.62 ± 0.25 (6)	10.2 ± 0.29 (5)	8.72 ± 0.24 (7)	8.54 ± 0.26 (5)	9.70 ± 0.26 (5)	8.09 ± 0.25 (5)	8.78 ± 0.27 (5)	7.77 ± 0.28 (5)	7.49 ± 0.20 (5)	7.10 ± 0.30 (5)	6.95 ± 0.37 (5)
Y299A	8.68 ± 0.32 (5)	7.97 ± 0.46 (6)	7.81 ± 0.38 (5)	10.4 ± 0.31 (5)	8.04 ± 0.36^*^ (6)	9.05 ± 0.38 (5)	9.35 ± 0.24 (6)	9.20 ± 0.40 (7)	8.85 ± 0.40 (5)	7.36 ± 0.10 (4)	−7.15 ± 0.36 (5)	7.32 ± 0.19 (5)	N.D.
I300A	9.31 ± 0.28 (5)	7.97 ± 0.32 (7)	8.55 ± 0.31 (5)	10.2 ± 0.29 (5)	N.D.	9.29 ± 0.36^*^ (5)	9.72 ± 0.26 (5)	N.D.	9.29 ± 0.31 (5)	7.18 ± 0.33 (5)	7.38 ± 0.40 (5)	7.54 ± 0.24 (5)	7.03 ± 0.35 (5)
F356A	8.05 ± 0.35^*^ (5)	7.70 ± 0.30 (6)	7.58 ± 0.27 (4)	9.73 ± 0.21 (6)	9.05 ± 0.41 (6)	9.38 ± 0.21^*^ (5)	8.85 ± 0.29^*^ (4)	N.D.	8.23 ± 0.33 (4)	7.09 ± 0.32 (4)	6.95 ± 0.45 (5)	6.65 ± 0.21^*^ (5)	6.65 ± 0.34 (4)
V357A	8.43 ± 0.25^*^ (6)	7.37 ± 0.48^*^ (5)	7.74 ± 0.26 (4)	10.1 ± 0.21 (6)	8.69 ± 0.42 (5)	8.44 ± 0.19 (5)	9.73 ± 0.32 (4)	7.69 ± 0.40^*^ (5)	8.19 ± 0.25 (4)	7.36 ± 0.33 (5)	7.29 ± 0.44 (5)	7.29 ± 0.15 (5)	N.D.
V358A	9.29 ± 0.28 (4)	7.28 ± 0.17^*^ (6)	7.31 ± 0.13^*^ (5)	10.7 ± 0.25 (4)	8.31 ± 0.13^*^ (6)	8.19 ± 0.13 (4)	9.40 ± 0.28 (4)	8.20 ± 0.26 (5)	8.38 ± 0.20 (4)	7.50 ± 0.33 (4)	7.06 ± 0.30 (5)	7.42 ± 0.27 (5)	7.34 ± 0.50 (5)
F359A	8.50 ± 0.21^*^ (5)	7.08 ± 0.24^*^ (5)	7.07 ± 0.15^*^ (5)	10.6 ± 0.20 (5)	8.29 ± 0.14^*^ (5)	7.79 ± 0.13^*^ (5)	9.65 ± 0.28 (4)	7.68 ± 0.24^*^ (5)	7.87 ± 0.18^*^ (5)	7.33 ± 0.31 (5)	7.10 ± 0.31 (4)	7.49 ± 0.19 (5)	6.64 ± 0.29 (5)
P360A	7.96 ± 0.34^*^ (5)	6.77 ± 0.26^*^ (5)	6.98 ± 0.40^*^ (5)	10.0 ± 0.20 (5)	7.99 ± 0.13^*^ (6)	7.50 ± 0.15^*^ (5)	9.03 ± 0.27^*^ (4)	6.76 ± 0.28^*^ (5)	7.31 ± 0.22^*^ (5)	7.24 ± 0.28 (5)	N.D.	7.59 ± 0.34 (5)	N.D.
W361A	8.69 ± 0.23 (5)	7.55 ± 0.23^*^ (6)	7.30 ± 0.20^*^ (5)	10.4 ± 0.21 (5)	8.66 ± 0.15 (5)	8.16 ± 0.15 (4)	9.10 ± 0.31 (4)	7.80 ± 0.25^*^ (6)	7.99 ± 0.27 (5)	7.40 ± 0.28 (5)	7.23 ± 0.44 (4)	6.73 ± 0.23^*^ (5)	6.90 ± 0.38 (5)
R362A	8.22 ± 0.24^*^ (4)	7.34 ± 0.27^*^ (6)	7.49 ± 0.29 (5)	9.85 ± 0.23 (4)	8.51 ± 0.25 (5)	8.53 ± 0.16 (4)	8.45 ± 0.32^*^ (4)	7.68 ± 0.42^*^ (6)	7.91 ± 0.25^*^ (5)	7.27 ± 0.38 (4)	6.78 ± 0.54 (4)	7.62 ± 0.10 (5)	7.01 ± 0.61 (5)
P363A	8.29 ± 0.25* (5)	N.D.	7.38 ± 0.30 (5)	10.4 ± 0.20 (5)	9.04 ± 0.19 (5)	8.57 ± 0.18 (4)	8.68 ± 0.30^*^ (4)	8.08 ± 0.25 (6)	8.05 ± 0.25 (5)	7.48 ± 0.27 (6)	7.43 ± 0.63 (4)	7.25 ± 0.16 (5)	N.D.
S364A	9.56 ± 0.21 (5)	7.47 ± 0.22^*^ (5)	7.82 ± 0.14 (5)	10.5 ± 0.21 (5)	8.56 ± 0.18 (5)	8.23 ± 0.17 (4)	10.2 ± 0.35 (4)	−8.00 ± 0.20 (5)	8.11 ± 0.17 (4)	8.18 ± 0.33 (4)	7.76 ± 0.23 (4)	7.53 ± 0.31 (4)	7.75 ± 0.30 (4)
N365A	9.55 ± 0.22 (5)	8.08 ± 0.28 (5)	7.93 ± 0.18 (5)	10.6 ± 0.24 (5)	8.71 ± 0.26 (4)	8.57 ± 0.20 (4)	10.4 ± 0.27 (4)	8.49 ± 0.31 (5)	8.58 ± 0.18 (5)	7.93 ± 0.30 (4)	7.56 ± 0.26 (5)	7.41 ± 0.15 (5)	7.30 ± 0.36 (4)
K366A	9.84 ± 0.31 (5)	8.20 ± 0.17 (5)	7.52 ± 0.14 (5)	10.6 ± 0.24 (5)	8.73 ± 0.17 (5)	8.41 ± 0.13 (4)	10.5 ± 0.29 (4)	8.68 ± 0.21 (5)	8.17 ± 0.13 (5)	7.83 ± 0.20 (4)	7.66 ± 0.17 (5)	8.02 ± 0.34 (5)	6.89 ± 0.16 (4)
M367A	9.49 ± 0.34 (5)	7.50 ± 0.17^*^ (5)	7.91 ± 0.14 (5)	10.8 ± 0.25 (5)	8.27 ± 0.13^*^ (6)	8.28 ± 0.14 (4)	10.2 ± 0.31 (4)	8.02 ± 0.19 (5)	7.93 ± 0.14^*^ (5)	8.09 ± 0.23 (4)	7.04 ± 0.24 (5)	7.27 ± 0.23 (5)	6.77 ± 0.27 (4)
L368A	9.85 ± 0.32 (5)	7.41 ± 0.17^*^ (6)	7.68 ± 0.16 (4)	10.4 ± 0.20 (5)	8.51 ± 0.17 (5)	8.21 ± 0.13 (4)	9.63 ± 0.33 (4)	7.66 ± 0.22^*^ (5)	8.21 ± 0.14 (5)	7.61 ± 0.28 (5)	7.35 ± 0.21 (4)	7.67 ± 0.32 (5)	6.94 ± 0.24 (4)
G369A	9.31 ± 0.22 (5)	7.28 ± 0.21^*^ (5)	7.78 ± 0.13 (5)	10.4 ± 0.24 (5)	8.61 ± 0.16 (5)	8.22 ± 0.16 (4)	9.97 ± 0.30 (4)	7.80 ± 0.19 (5)	7.90 ± 0.17^*^ (5)	7.56 ± 0.28 (4)	7.55 ± 0.26 (4)	7.06 ± 0.25 (5)	6.84 ± 0.23 (4)
K370A	N.D.	N.D.	N.D.	N.D.	N.D.	N.D.	N.D.	N.D.	N.D.	N.D.	N.D.	N.D.	N.D.
I371A	9.63 ± 0.33 (5)	8.07 ± 0.22 (5)	N.D.	10.8 ± 0.21 (5)	8.45 ± 0.19 (6)	N.D.	10 ± 0.30 (4)	8.58 ± 0.19 (5)	N.D.	8.29 ± 0.26 (5)	N.D.	7.02 ± 0.34 (5)	N.D.
Y372A	7.18 ± 0.35^*^ (4)	7.67 ± 0.35 (5)	6.76 ± 0.30^*^ (5)	9.60 ± 0.22 (4)	8.81 ± 0.43 (5)	8.55 ± 0.42 (5)	8.23 ± 0.35^*^ (4)	8.27 ± 0.34 (5)	7.57 ± 0.28^*^ (4)	N.D.	N.D.	6.93 ± 0.25 (5)	N.D.
D373A	8.41 ± 0.27^*^ (4)	7.51 ± 0.20^*^ (5)	7.41 ± 0.27 (5)	10.3 ± 0.21 (4)	8.60 ± 0.22 (5)	8.34 ± 0.15 (4)	8.39 ± 0.34^*^ (4)	7.59 ± 0.28^*^ (5)	8.09 ± 0.28 (4)	7.81 ± 0.29 (5)	N.D.	7.72 ± 0.24 (5)	N.D.
Y374A	9.45 ± 0.28 (5)	7.32 ± 0.20^*^ (5)	7.67 ± 0.18 (5)	10.5 ± 0.20 (5)	8.34 ± 0.15^*^ (6)	8.36 ± 0.18 (4)	10.4 ± 0.32 (4)	7.60 ± 0.18^*^ (5)	8.35 ± 0.20 (4)	7.75 ± 0.33 (4)	7.12 ± 0.3 (4)	7.35 ± 0.34 (5)	7.07 ± 0.37 (5)
V375A	9.21 ± 0.20 (5)	7.98 ± 0.28 (5)	7.88 ± 0.28 (4)	10.2 ± 0.20 (5)	8.32 ± 0.22^*^ (6)	8.70 ± 0.23 (5)	9.59 ± 0.41 (4)	8.25 ± 0.31 (5)	8.32 ± 0.23 (5)	7.79 ± 0.29 (5)	6.70 ± 0.26 (4)	7.27 ± 0.26 (5)	6.78 ± 0.40 (5)
M376A	8.12 ± 0.31^*^ (5)	7.83 ± 0.41 (4)	7.47 ± 0.21 (4)	10.2 ± 0.21 (5)	8.43 ± 0.19 (4)	8.17 ± 0.25 (4)	9.30 ± 0.36 (4)	7.90 ± 0.30 (5)	8.54 ± 0.14 (5)	7.09 ± 0.27 (5)	7.09 ± 0.43 (5)	6.63 ± 0.31^*^ (6)	7.52 ± 0.26 (5)

For each receptor mutant and ligand, concentration-response data for each pathway were fit with the Black and Leff operational model to derive a functional affinity (pK_A_) for each pathway. All values are mean ± S.E.M. (independent “n” values are indicated within parentheses). For each ligand, significance of changes was determined by comparison of mutant receptor pK_A_ to WT receptor pK_A_ in a one-way analysis of variance followed by Dunnett’s post-test (p < 0.05 represented by *). (N.D.) data were not able to be reliably determined. WT pK_A_ values for sCT were 10.52 ± 0.09, 9.20 ± 0.12, and 8.55 ± 0.07, for cAMP, IP1 and pERK signalling, respectively. The corresponding values for hCT were 9.54 ± 0.09, 8.54 ± 0.10, and 8.15 ± 0.08, and for pCT were 10.12 ± 0.10, 8.75 ± 0.13 and 8.74 ± 0.09. pK_A_ values for cAMP and pERK were, respectively, 7.50 ± 0.33 and 7.23 ± 0.11 for rAmy, and 7.69 ± 0.16 and 7.10 ± 0.11 for CGRP.

**Fig. 9:**
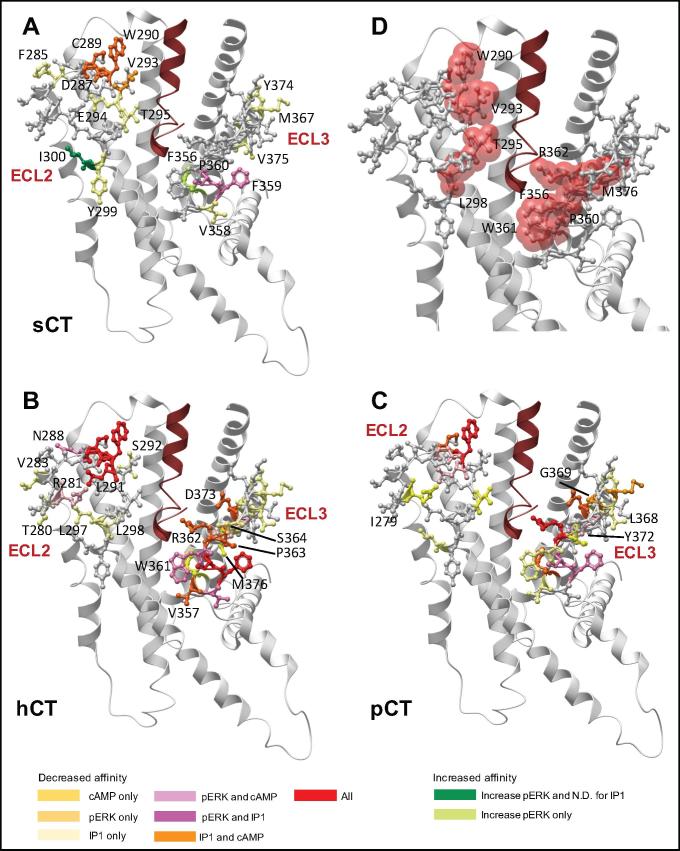
Mutants that differentially alter peptide-dependent functional affinity cluster into subdomains in ECL2 and ECL3. (A-C) Mutants that alter functional affinity are displayed in x-stick representation, coloured according to effect on the different signalling pathways. (A) Effect of mutation on sCT, (B) hCT, or (C) pCT pK_A_. Mutants that did not significantly affect functional affinity in any pathway are coloured grey. The full receptor TM domain is displayed as grey ribbon, and sCT in dark red ribbon, with the receptor ECD, and peptide C-terminal residues omitted for clarity. (D) ECL2 and ECL3 amino acids within 5 Å of sCT in the model are displayed in red transparent cpk representation. (For interpretation of the references to colour in this figure legend, the reader is referred to the web version of this article.)

There were distinct patterns in the effect of mutation on pK_A_ values both across pathways and between peptides. sCT functional affinities were the least sensitive to mutation, and in particular, mutations in ECL3 had limited impact. This contrasts with hCT and pCT where ECL3 mutations had widespread effect with greatest impact on pK_A_ values derived from IP1 and cAMP signalling (Fig. 2, Fig. 8, Fig. 9, Table 5).

The F359A and P360A mutants induced a global decrease in functional affinity for pERK and IP1 pathways, but had differential effect on cAMP responses. Neither affected sCT, while P360A attenuated pK_A_ values for both hCT and pCT, with a selective loss of affinity for hCT observed for the F359A mutant (Fig. 2, Fig. 8, Fig. 9, Table 5).

For sCT, there was a selective decrease in pK_A_ values linked to pERK for the V293A mutant and a selective increase for the I300A and F356A mutants, while none of the other mutants altered pERK-derived pK_A_ values (Table 5). Analysis of IP1 signalling revealed an IP1 specific loss of affinity for all 3 peptides at the Y374A mutant, while there was preferential loss of affinity for sCT and hCT for the V358A and M367A mutants. For the latter mutant, while there was no significant effect on pCT functional affinity linked to IP1 signalling, there was a selective decrease in the pERK linked pK_A_ value. There was also a selective decrease in sCT IP1-derived functional affinity for the F285A, E294A, T295A, and Y299A mutants. No other mutants altered sCT functional affinities for any of the pathways studied (Fig. 2, Fig. 8, Fig. 9, Table 5).

hCT and pCT displayed intriguing differences in the pattern of mutational effect on pK_A_ values derived from analysis of IP1 and pERK responses. For hCT there was only limited effect of mutation in ECL3 on pERK-derived pK_A_ values and these were confined to the membrane proximal ECL3/TM6 boundary (V358A-R362A) and Y372A that packs up against this region in the WT receptor (Fig. 2, Fig. 8, Fig. 9, Table 5). This segment was also important for pCT pERK-derived functional affinity, but additional effect was seen with the M367A-G369A mutants that are involved with packing at the top of the extension of TM7, and which are important for IP1-derived functional affinity of hCT and pCT, albeit that not all effects reached significance for both peptides (Table 5). ECL3 was important for IP1-derived functional affinities for both hCT and pCT, with those mutations impacting on pCT also decreasing hCT pK_A_ values, including residues at the ECL3/TM6 boundary (F356A-R362A), residues in the distal part of ECL3 that are likely important for TM7/TM1 packing (D373A, Y374A) as well as residues located in the upper extension of TM7 (M368A-G369A) (Fig. 2, Fig. 8, Fig. 9, Table 5). However, the network of residues important for hCT pK_A_ values was more extensive for both the TM6/ECL3 boundary (P363A, S364A), and the extension of TM7 (M367A). In contrast, ECL2 played only a limited role in pCT functional affinity derived from either IP1 or pERK signalling. Only the I279A mutant altered pCT IP1-derived pK_A_ values, although no quantifiable response was obtained for the poorly expressed C289A, W290A and I300A mutants (Table 5). For pERK functional affinity, only the D287A, W290A and L291A mutants attenuated pCT affinity. This central network was also important of hCT pERK-derived pK_A_ values, with N288A and C289A also reducing hCT functional affinity. It is likely that the R281A mutant additionally attenuates hCT affinity as no quantifiable response was detected, and there was a large decrease in cAMP-derived pK_A_. For hCT functional affinity derived from IP1 signalling, there was a broader impact of mutation for ECL2 residues that included T280A-V283A, residues of the central core and boundary with ECL1 (D287A-S292A), and L297A and L298A (Fig. 2, Fig. 8, Fig. 9, Table 5).

**Fig. 10:**
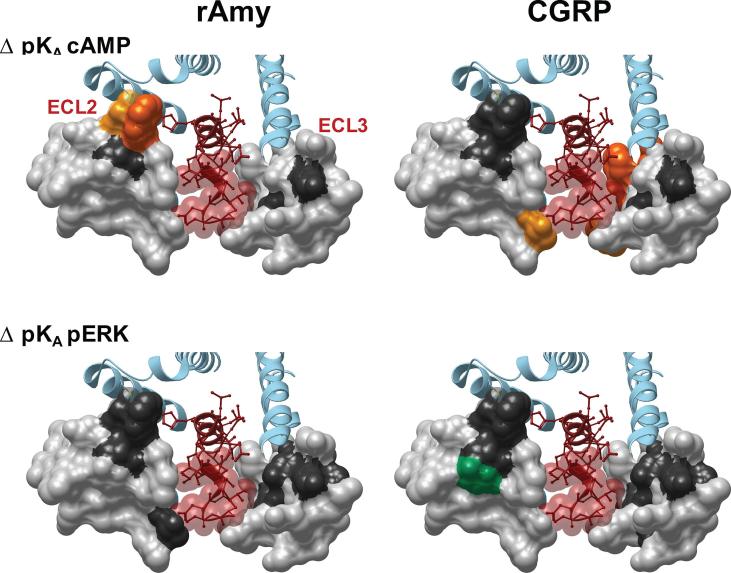
Alanine mutation of ECL2 and ECL3 of the hCTR alters amylin and CGRP functional affinity (pK_A_) in a peptide- and pathway-specific manner. Functional affinities derived from operation fitting of concentration-response curves in cAMP accumulation (upper panel) and pERK (lower panel) are displayed as ΔpK_A_. Illustrated is a top view of the active, sCT-bound, hCTR model with ECL2 and ECL3 shown in surface representation. Mutations that significantly alter peptide affinity are coloured according the magnitude of effect (from Table 5), with mutated amino acids without significant alteration to affinity coloured grey. sCT is shown as dark red, with side chains in proximity to the ECLs displayed in x-stick, and residues 1–7 that are critical for receptor activation displayed in transparent cpk. (For interpretation of the references to colour in this figure legend, the reader is referred to the web version of this article.)

Due to the low affinity of amylin and CGRP for CTR, and poor coupling to IP1 signalling (data not shown), no competitive binding data or IP1-derived functional affinity data could be obtained for these peptides. pK_A_ values from operational analysis of the cAMP signalling could be derived for most mutants, revealing only limited impact on either amylin or CGRP functional affinity. This was particularly true for amylin that mirrored the observations for sCT, with ∼10-fold loss of affinity for the C289A and W290A mutants. No quantifiable response for the D287A and Y372A mutants (that had weak reductions in sCT functional affinity) was observed, while all other mutants failed to significantly alter affinity (Fig. 8; Table 5, Fig. 6). Although the magnitude of effect was also limited for CGRP in this pathway, there were broader effects of mutations, including loss of detectable response for D287A, C289A, and W290A, and reduced affinity for L291A, T295A, F356A, W361A and M376A. These residues were also important for hCT functional affinity at this pathway (Fig. 3, Fig. 10; Table 5).

Both amylin and CGRP are only weakly coupled to pERK, and alanine mutation either had no effect or attenuated responses such that they there were not quantifiable. Nonetheless, interesting differences were observed between the effect on amylin and CGRP signalling. Residues with very low expression, including D287A, C289A, W290A and I371A had no detectable response for either peptide. Loss of response for both peptides was also seen for the P360A, Y372A and D373A mutants, despite similar or greater (P360A) cell surface expression of the receptor. The L291A, and Y299A mutants selectively attenuated, and the N286A mutant selectively enhanced, CGRP functional affinity. Functional CGRP affinity for T295A was not significantly altered, and not determined for amylin due to large error in parameter estimates, despite a measurable response (Fig. 8, Fig. 10; Table 5).

### 3.4 ECL2 and ECL3 play distinct roles in pathway specific efficacy

#### 3.4.1 Calcitonin peptides

**Fig. 11:**
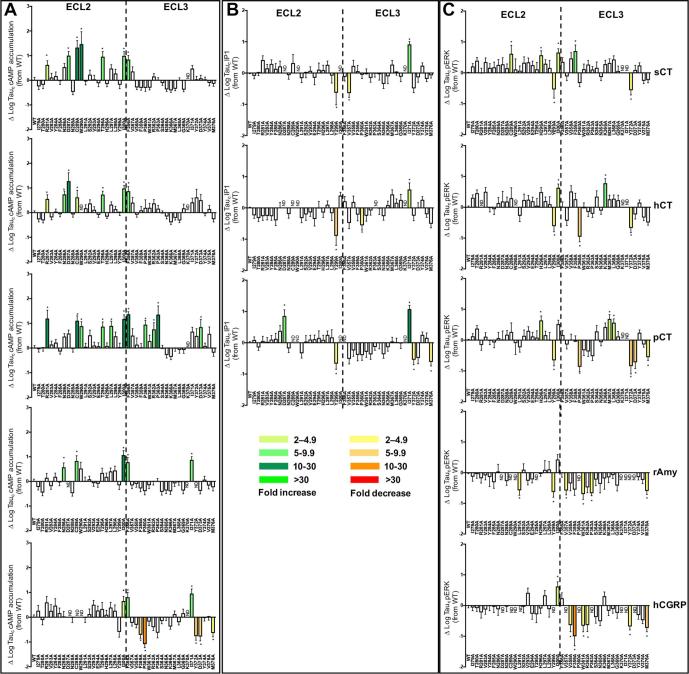
Alanine mutation of ECL2 and ECL3 of the hCTR selectively alters peptide efficacy. Concentration-response data for each peptide in (A) cAMP accumulation, (B) IP1 accumulation, or (C) pERK assays were fit to the operational model of agonism to calculate the affinity-independent coupling efficacy log(τ) of each receptor (mutant or WT) for each signalling pathway. The log(τ) values of each receptor were then corrected for expression to obtain log(τc). Graphs show the differences relative to WT. All values are mean ± S.E.M. of 4–11 (WT 25–47) independent experiments conducted in duplicate. Significance of changes were determined by comparison of the WT to the other receptor mutants in a one-way analysis of variance and Dunnett’s post-test of determined log(τc) values (Table 2) with significant changes (P < 0.05) denoted by *, and coloured according to the magnitude of effect. N.D., efficacy not determined.

**Fig. 12:**
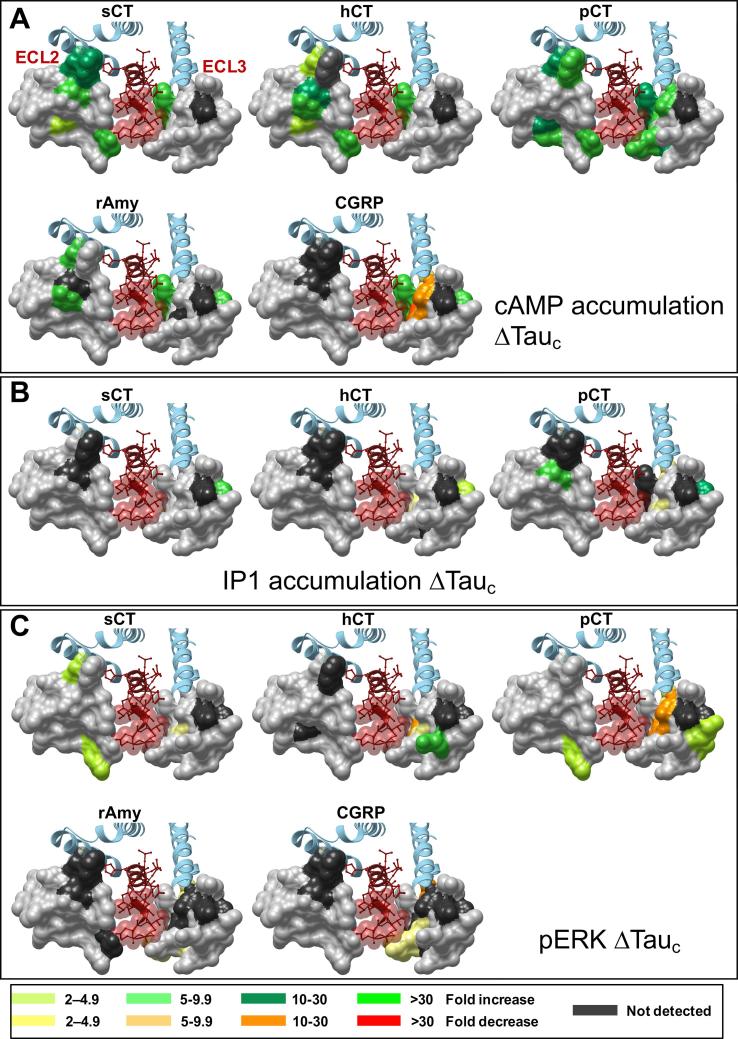
Alanine mutation of ECL2 and ECL3 of the hCTR differentially modulates CT and related peptide efficacy in a pathway-dependent manner. Illustrated are top views of the active, sCT-bound, hCTR model with ECL2 and ECL3 shown in surface representation. Mutations that significantly alter peptide efficacy are coloured according the magnitude of effect (from Table 6 and Fig. 11), with mutated amino acids without significant alteration to efficacy coloured grey. Salmon CT is shown as dark red, with side chains in proximity to the ECLs displayed in x-stick, and residues 1–7 that are critical for receptor activation display in transparent cpk. (A) Changes to cAMP efficacy. (B) Changes to IP1 efficacy. (C) Changes to pERK efficacy. (For interpretation of the references to colour in this figure legend, the reader is referred to the web version of this article.)

Interestingly, despite detrimental effects on functional affinity, only enhancement of cAMP efficacy was observed for any of the CT peptides (Figs. 11A and 12A; Table 6). For sCT and hCT, the effect of mutation was similar, and confined to ECL2, with the exception of F356A that resides at the base of the peptide binding pocket. Enhanced efficacy was seen for both sCT and hCT for R281A, D287A, C289A, W290A, T295A and I300A (Fig. 11A and 12A; Table 6). These mutants also had reduced cell surface expression (Fig. 1), indicative of destabilization of the receptor in a manner that lowers the barrier to Gs coupling. N286A caused a selective enhancement of hCT efficacy, while pCT had both overlapping and distinct effects following receptor mutation. In ECL2, the effect of mutation was conserved with the other peptides except that there was no change in pCT efficacy with the D287A mutant, and a selective enhancement of efficacy at the L297A mutant. Strikingly, ECL3 residues were also important for pCT cAMP efficacy, with P360A, R362A, P363A and D373A causing selective enhancement of efficacy (Fig. 11A and 12A; Table 6).

**Table 6:** Effect of single alanine mutation in hCTR ECL2 or ECL3 on efficacy of CT-family peptides.

	hCT			sCT			pCT			rAmy		hαCGRP	
	cAMP	IP1	pERK1/2	cAMP	IP1	pERK1/2	cAMP	IP1	pERK1/2	cAMP	pERK1/2	cAMP	pERK1/2
WT	0.18 ± 0.07 (36)	−0.07 ± 0.02 (47)	−0.11 ± 0.04 (34)	0.25 ± 0.12 (36)	−0.04 ± 0.02 (44)	−0.15 ± 0.02 (35)	0.12 ± 0.10 (31)	−0.07 ± 0.04 (37)	−0.11 ± 0.03 (32)	0.12 ± 0.09 (31)	−0.10 ± 0.03 (30)	0.11 ± 0.09 (25)	−0.08 ± 0.08 (30)
I279A	−0.09 ± 0.10 (4)	−0.30 ± 0.10 (7)	0.20 ± 0.09 (5)	0.00 ± 0.13 (5)	−0.13 ± 0.10 (7)	0.05 ± 0.09 (5)	0.07 ± 0.12 (6)	0.01 ± 0.10 (5)	0.02 ± 0.08 (5)	−0.11 ± 0.12 (5)	−0.24 ± 0.15 (5)	0.37 ± 0.22 (5)	−0.14 ± 0.11 (5)
T280A	−0.13 ± 0.10 (4)	−0.39 ± 0.11 (6)	0.35 ± 0.11 (6)	0.06 ± 0.16 (4)	−0.01 ± 0.11 (7)	0.24 ± 0.10 (6)	0.14 ± 0.15 (5)	−0.22 ± 0.10 (7)	0.27 ± 0.10 (6)	−0.34 ± 0.13 (4)	−0.15 ± 0.14 (6)	−0.01 ± 0.20 (5)	−0.17 ± 0.11 (6)
R281A	0.74 ± 0.17* (8)	−0.33 ± 0.14 (7)	N.D.	0.87 ± 0.17* (11)	0.37 ± 0.14 (6)	−0.16 ± 0.16 (5)	1.31 ± 0.34* (6)	−0.02 ± 0.15 (6)	−0.27 ± 0.17 (5)	0.26 ± 0.18 (5)	−0.29 ± 0.16 (5)	0.71 ± 0.24 (5)	−0.30 ± 0.19 (5)
V283A	−0.01 ± 0.10 (4)	−0.32 ± 0.13 (5)	0.40 ± 0.14 (5)	0.36 ± 0.17 (5)	0.11 ± 0.13 (7)	0.20 ± 0.13 (6)	0.26 ± 0.14 (5)	−0.04 ± 0.13 (7)	0.00 ± 0.13 (5)	−0.07 ± 0.13 (5)	−0.19 ± 0.15 (5)	0.36 ± 0.25 (5)	−0.21 ± 0.15 (4)
Y284A	0.22 ± 0.10 (5)	−0.33 ± 0.13 (7)	−0.12 ± 0.14 (5)	0.42 ± 0.16 (5)	0.25 ± 0.13 (6)	0.01 ± 0.13 (5)	0.33 ± 0.13 (6)	0.03 ± 0.13 (7)	−0.12 ± 0.13 (5)	0.30 ± 0.15 (5)	−0.24 ± 0.32 (5)	0.57 ± 0.28 (5)	−0.09 ± 0.16 (5)
F285A	0.12 ± 0.10 (5)	−0.47 ± 0.17 (6)	−0.17 ± 0.17 (5)	0.23 ± 0.15 (5)	0.09 ± 0.17 (5)	0.03 ± 0.17 (5)	−0.02 ± 0.11 (6)	0.05 ± 0.18 (6)	−0.18 ± 0.17 (5)	0.08 ± 0.14 (5)	−0.42 ± 0.20 (5)	0.27 ± 0.21 (5)	−0.12 ± 0.19 (5)
N286A	0.92 ± 0.14* (5)	−0.07 ± 0.17 (6)	0.03 ± 0.18 (5)	0.78 ± 0.15 (5)	0.17 ± 0.17 (5)	0.10 ± 0.18 (5)	0.58 ± 0.12 (5)	0.30 ± 0.17 (7)	−0.04 ± 0.18 (5)	0.69 ± 0.18* (5)	−0.07 ± 0.24 (5)	0.13 ± 0.21 (5)	−0.20 ± 0.20 (5)
D287A	1.46 ± 0.34* (6)	N.D.	0.08 ± 0.33 (5)	1.25 ± 0.17* (10)	N.D.	0.12 ± 0.21 (5)	0.70 ± 0.14 (6)	0.78 ± 0.20* (6)	−0.10 ± 0.29 (5)	N.D.	N.D.	N.D.	N.D.
N288A	0.11 ± 0.12 (5)	−0.26 ± 0.13 (7)	0.08 ± 0.15 (5)	−0.23 ± 0.11 (6)	−0.11 ± 0.13 (6)	0.00 ± 0.14 (5)	0.09 ± 0.14 (5)	−0.25 ± 0.14 (7)	0.07 ± 0.14 (5)	−0.36 ± 0.12 (5)	−0.38 ± 0.14 (5)	0.10 ± 0.22 (5)	−0.12 ± 0.16 (5)
C289A	0.80 ± 0.28* (8)	N.D.	0.19 ± 0.34 (5)	1.57 ± 0.29* (10)	0.27 ± 0.27 (6)	0.46 ± 0.28* (5)	1.22 ± 0.20* (8)	N.D.	0.16 ± 0.29 (5)	0.95 ± 0.21* (5)	N.D.	N.D.	N.D.
W290A	N.D.	N.D.	N.D.	1.71 ± 0.50* (11)	N.D.	−0.04 ± 0.34 (5)	1.01 ± 0.18* (5)	N.D.	−0.20 ± 0.37 (5)	0.64 ± 0.25 (5)	N.D.	N.D.	N.D.
L291A	0.36 ± 0.14 (10)	−0.39 ± 0.15 (7)	−0.38 ± 0.19 (5)	0.20 ± 0.14 (10)	−0.21 ± 0.15 (7)	−0.08 ± 0.15 (5)	0.17 ± 0.14 (6)	−0.41 ± 0.15 (7)	−0.34 ± 0.16 (5)	0.13 ± 0.16 (5)	−0.68 ± 0.16* (5)	0.09 ± 0.20 (5)	N.D.
S292A	0.56 ± 0.15 (6)	−0.28 ± 0.19 (6)	0.13 ± 0.19 (5)	0.48 ± 0.18 (6)	−0.01 ± 0.19 (7)	0.17 ± 0.19 (5)	0.64 ± 0.21 (6)	−0.04 ± 0.19 (7)	0.03 ± 0.19 (5)	0.20 ± 0.13 (6)	−0.01 ± 0.19 (6)	0.23 ± 0.21 (5)	−0.12 ± 0.20 (5)
V293A	0.30 ± 0.12 (5)	−0.21 ± 0.13 (6)	0.14 ± 0.14 (5)	0.28 ± 0.17 (5)	0.03 ± 0.13 (7)	0.19 ± 0.14 (5)	0.22 ± 0.14 (6)	0.03 ± 0.14 (5)	0.19 ± 0.13 (5)	0.05 ± 0.13 (6)	−0.37 ± 0.14 (5)	0.61 ± 0.17 (5)	0.33 ± 0.15 (5)
E294A	0.10 ± 0.10 (5)	−0.42 ± 0.23 (6)	0.09 ± 0.13 (5)	0.13 ± 0.14 (5)	−0.20 ± 0.23 (6)	0.10 ± 0.13 (5)	0.19 ± 0.14 (5)	0.04 ± 0.13 (6)	0.01 ± 0.13 (5)	0.02 ± 0.14 (5)	−0.32 ± 0.12 (5)	0.38 ± 0.18 (5)	−0.35 ± 0.18 (5)
T295A	0.91 ± 0.14* (5)	−0.08 ± 0.21 (6)	−0.05 ± 0.23 (5)	1.20 ± 0.21* (5)	0.06 ± 0.21 (6)	−0.04 ± 0.22 (5)	0.99 ± 0.19* (5)	0.08 ± 0.23 (6)	−0.03 ± 0.22 (5)	0.46 ± 0.20 (5)	N.D.	0.44 ± 0.27 (5)	−0.37 ± 0.27 (5)
H296A	0.02 ± 0.08 (10)	−0.21 ± 0.16 (7)	0.39 ± 0.15 (5)	0.10 ± 0.11 (10)	0.05 ± 0.16 (7)	0.42 ± 0.15* (5)	0.20 ± 0.13 (5)	0.02 ± 0.14 (6)	0.53 ± 0.14* (6)	0.30 ± 0.15 (5)	−0.14 ± 0.08 (5)	0.21 ± 0.18 (6)	−0.18 ± 0.16 (5)
L297A	0.51 ± 0.11 (4)	0.04 ± 0.12 (7)	0.09 ± 0.13 (5)	0.72 ± 0.15 (4)	0.22 ± 0.12 (6)	0.14 ± 0.13 (5)	1.01 ± 0.19* (6)	0.17 ± 0.13 (7)	0.05 ± 0.13 (5)	0.52 ± 0.14 (5)	−−0.01 ± 0.30 (5)	0.50 ± 0.21 (5)	0.25 ± 0.15 (5)
L298A	0.33 ± 0.11 (5)	−0.23 ± 0.24 (7)	−0.01 ± 0.24 (5)	0.52 ± 0.17 (5)	−0.13 ± 0.24 (7)	0.00 ± 0.24 (5)	0.59 ± 0.17 (5)	0.10 ± 0.24 (5)	−0.10 ± 0.24 (5)	0.56 ± 0.17 (5)	0.00 ± 0.25 (5)	0.37 ± 0.23 (5)	−0.20 ± 0.26 (5)
Y299A	0.22 ± 0.15 (5)	−0.96 ± 0.31* (6)	−0.72 ± 0.18* (5)	0.07 ± 0.16 (5)	−0.68 ± 0.39* (6)	−0.69 ± 0.28* (5)	0.15 ± 0.16 (6)	−0.74 ± 0.18* (7)	−0.76 ± 0.18* (5)	−0.04 ± 0.17 (4)	−0.73 ± 0.17* (5)	−0.49 ± 0.21 (5)	N.D.
I300A	1.16 ± 0.12* (5)	0.33 ± 0.10 (7)	0.53 ± 0.12* (5)	1.24 ± 0.17* (5)	N.D.	0.50 ± 0.12* (5)	1.3 ± 0.17* (5)	N.D.	0.41 ± 0.13 (5)	1.19 ± 0.18* (5)	0.33 ± 0.16 (5)	0.76 ± 0.19* (5)	0.54 ± 0.15* (5)
F356A	1.05 ± 0.19* (5)	0.15 ± 0.15 (6)	−0.10 ± 0.17 (4)	1.08 ± 0.17* (6)	−0.16 ± 0.14 (6)	0.20 ± 0.12 (5)	1.49 ± 0.10* (4)	N.D.	0.06 ± 0.15 (4)	0.90 ± 0.19* (4)	−0.12 ± 0.14 (5)	0.91 ± 0.2* (5)	0.16 ± 0.18 (4)
V357A	0.59 ± 0.21 (6)	−0.55 ± 0.18 (5)	−0.54 ± 0.16 (4)	0.61 ± 0.18 (6)	−0.69 ± 0.14* (5)	−0.27 ± 0.14 (5)	0.62 ± 0.28 (4)	−0.59 ± 0.16 (5)	−0.25 ± 0.15 (4)	−0.05 ± 0.13 (5)	−0.69 ± 0.14* (5)	−0.12 ± 0.13 (5)	N.D.
V358A	0.10 ± 0.10 (4)	0.12 ± 0.19 (6)	0.41 ± 0.19 (5)	−0.04 ± 0.09 (4)	0.18 ± 0.18 (6)	0.31 ± 0.18 (4)	0.25 ± 0.27 (4)	−0.32 ± 0.19 (5)	−0.10 ± 0.19 (4)	0.02 ± 0.14 (4)	−0.45 ± 0.24 (5)	−0.20 ± 0.12 (5)	−0.72 ± 0.22* (5)
F359A	0.28 ± 0.16 (5)	−0.28 ± 0.20 (5)	0.15 ± 0.19 (5)	−0.06 ± 0.09 (5)	0.01 ± 0.19 (5)	0.55 ± 0.20* (5)	0.14 ± 0.16 (4)	−0.46 ± 0.19 (5)	−0.16 ± 0.19 (5)	−0.17 ± 0.13 (5)	−0.66 ± 0.19 (4)	−0.60 ± 0.14* (5)	−1.08 ± 0.31* (5)
P360A	0.38 ± 0.23 (5)	−0.62 ± 0.15* (5)	−1.06 ± 0.16* (5)	−0.06 ± 0.14 (5)	−0.10 ± 0.12 (6)	−0.48 ± 0.12 (5)	1.07 ± 0.17* (4)	−0.47 ± 0.17 (5)	−0.98 ± 0.16* (5)	−0.28 ± 0.15 (5)	N.D.	−0.98 ± 0.14* (5)	N.D.
W361A	0.38 ± 0.18 (5)	−0.32 ± 0.17 (6)	−0.14 ± 0.17 (5)	−0.05 ± 0.09 (5)	−0.08 ± 0.17 (5)	−0.03 ± 0.17 (4)	0.4 ± 0.24 (4)	−0.36 ± 0.17 (6)	−0.44 ± 0.17 (5)	−0.04 ± 0.13 (5)	−0.80 ± 0.18* (4)	−0.07 ± 0.16 (5)	−0.74 ± 0.20* (5)
R362A	0.56 ± 0.15 (4)	−0.15 ± 0.15 (6)	−0.26 ± 0.15 (5)	0.40 ± 0.12 (4)	−0.20 ± 0.14 (5)	0.11 ± 0.13 (4)	0.87 ± 0.27* (4)	−0.44 ± 0.16 (6)	−0.48 ± 0.16 (5)	0.12 ± 0.15 (4)	−0.58 ± 0.18 (4)	−0.30 ± 0.14 (5)	−0.71 ± 0.21* (5)
P363A	0.34 ± 0.13 (5)	N.D.	−0.32 ± 0.12 (5)	0.26 ± 0.10 (5)	− 0.05 ± 0.12 (5)	−0.05 ± 0.1 (4)	1.47 ± 0.35* (4)	−0.21 ± 0.11 (6)	−0.63 ± 0.14 (5)	0.11 ± 0.12 (6)	−0.77 ± 0.08* (4)	−0.52 ± 0.21 (5)	N.D.
S364A	0.16 ± 0.09 (5)	−0.08 ± 0.19 (5)	0.24 ± 0.19 (5)	0.13 ± 0.09 (5)	−0.08 ± 0.19 (5)	0.01 ± 0.19 (4)	0.09 ± 0.14 (4)	−0.08 ± 0.19 (5)	0.17 ± 0.19 (4)	−0.31 ± 0.12 (4)	−0.33 ± 0.20 (4)	−0.04 ± 0.12 (4)	−0.46 ± 0.20 (4)
N365A	0.03 ± 0.10 (5)	−0.33 ± 0.13 (5)	−0.21 ± 0.12 (5)	−0.12 ± 0.08 (5)	−0.39 ± 0.15 (4)	−0.29 ± 0.12 (4)	−0.16 ± 0.13 (4)	−0.44 ± 0.13 (5)	−0.18 ± 0.12 (5)	−0.25 ± 0.13 (4)	−0.47 ± 0.29 (5)	0.11 ± 0.13 (5)	−0.59 ± 0.14 (4)
K366A	−0.21 ± 0.09 (5)	0.02 ± 0.13 (5)	0.67 ± 0.15* (5)	−0.16 ± 0.08 (5)	−0.15 ± 0.23 (5)	0.13 ± 0.13 (4)	−0.24 ± 0.11 (4)	−0.22 ± 0.13 (5)	0.32 ± 0.13 (5)	−0.28 ± 0.13 (4)	−0.29 ± 0.35 (5)	−0.27 ± 0.11 (5)	0.22 ± 0.14 (4)
M367A	−0.03 ± 0.09 (5)	0.36 ± 0.15 (4)	0.15 ± 0.13 (5)	−0.10 ± 0.08 (5)	0.22 ± 0.13 (6)	0.17 ± 0.13 (4)	0.11 ± 0.12 (4)	−0.03 ± 0.14 (5)	0.58 ± 0.14* (5)	0.04 ± 0.12 (4)	−0.26 ± 0.17 (5)	0.23 ± 0.13 (5)	−0.25 ± 0.16 (4)
L368A	−0.13 ± 0.09 (5)	0.10 ± 0.12 (6)	0.17 ± 0.12 (4)	0.25 ± 0.10 (5)	0.00 ± 0.12 (5)	0.28 ± 0.12 (4)	0.04 ± 0.13 (4)	−0.06 ± 0.13 (5)	0.46 ± 0.12* (5)	0.09 ± 0.12 (5)	−0.18 ± 0.12 (4)	−0.09 ± 0.11 (5)	−0.19 ± 0.14 (4)
G369A	0.36 ± 0.17 (5)	0.19 ± 0.18 (5)	0.12 ± 0.16 (5)	−0.13 ± 0.09 (5)	−0.16 ± 0.16 (5)	−0.09 ± 0.16 (4)	0.04 ± 0.13 (4)	−0.09 ± 0.16 (5)	0.01 ± 0.16 (5)	−0.25 ± 0.13 (4)	−0.53 ± 0.17 (4)	0.27 ± 0.17 (5)	−0.32 ± 0.17 (4)
K370A	N.D.	N.D.	N.D.	N.D.	N.D.	N.D.	N.D.	N.D.	N.D.	N.D.	N.D.	N.D.	N.D.
I371A	0.60 ± 0.17 (5)	0.52 ± 0.21* (7)	N.D.	0.72 ± 0.13 (5)	0.87 ± 0.09* (6)	N.D.	0.78 ± 0.18 (4)	1.00 ± 0.12* (5)	N.D.	0.99 ± 0.13* (5)	N.D.	1.06 ± 0.19* (5)	N.D.
Y372A	0.80 ± 0.33 (4)	−0.32 ± 0.16 (5)	−0.78 ± 0.15* (5)	0.29 ± 0.12 (4)	−0.53 ± 0.14 (5)	−0.72 ± 0.15* (4)	0.61 ± 0.29 (4)	−0.62 ± 0.16* (5)	−0.95 ± 0.25* (4)	N.D.	N.D.	−0.66 ± 0.16* (5)	N.D
D373A	0.69 ± 0.23 (4)	−0.06 ± 0.16 (5)	−0.34 ± 0.15 (5)	0.33 ± 0.12 (4)	−0.18 ± 0.16 (5)	−0.06 ± 0.15 (4)	0.96 ± 0.31* (4)	−0.56 ± 0.19 (5)	−0.83 ± 0.2* (5)	−0.27 ± 0.12 (5)	N.D.	−0.67 ± 0.18* (5)	N.D.
Y374A	0.15 ± 0.09 (5)	0.19 ± 0.10 (5)	0.05 ± 0.09 (5)	0.28 ± 0.09 (5)	0.19 ± 0.09 (6)	0.09 ± 0.09 (4)	0.2 ± 0.12 (4)	0.18 ± 0.09 (5)	0.13 ± 0.09 (4)	0.19 ± 0.14 (4)	−0.25 ± 0.09 (4)	0.08 ± 0.13 (5)	−0.39 ± 0.14 (5)
V375A	0.33 ± 0.15 (5)	−0.27 ± 0.10 (5)	−0.44 ± 0.10 (4)	0.14 ± 0.12 (5)	−0.23 ± 0.09 (6)	−0.41 ± 0.09 (5)	0.69 ± 0.28 (4)	0.08 ± 0.16 (5)	−0.25 ± 0.09 (5)	−0.05 ± 0.14 (5)	−0.34 ± 0.12 (5)	−0.15 ± 0.16 (5)	−0.56 ± 0.11 (5)
M376A	−0.08 ± 0.11 (5)	−0.57 ± 0.13 (4)	−0.60 ± 0.09 (4)	0.10 ± 0.10 (5)	−0.12 ± 0.09 (4)	−0.39 ± 0.09 (4)	−0.07 ± 0.15 (4)	−0.69 ± 0.13* (5)	−0.66 ± 0.11* (5)	−0.12 ± 0.15 (5)	−0.70 ± 0.12* (5)	−0.53 ± 0.16* (5)	−0.81 ± 0.13* (5)

For each receptor mutant and ligand, concentration-response data for each pathway were fit with the Black and Leff operational model to derive an affinity-independent measure of efficacy (log(τ)). These data were corrected for changes in cell surface expression from FACS to yield log(τc). All values are mean ± S.E.M. (independent “n” values are indicated within parentheses). For each ligand, significance of changes in log(τc) was determined by comparison of mutants to the WT receptor in a one-way analysis of variance followed by Dunnett’s post-test (p < 0.05 represented by *). (N.D.) data were not able to be reliably determined.

There was only limited impact of mutation on CT-mediated IP1 efficacy (Fig. 11B, Fig. 12B, Table 6). Within ECL2, no quantifiable response was seen with W290A for any of the peptides, with C289A for hCT and pCT (but no effect on sCT), and with D287A for sCT and hCT, but an enhanced efficacy for pCT with this mutation. The Y299A mutant attenuated efficacy for all peptides, while I300A abolished responses to sCT and pCT but not hCT (Table 6). Within ECL3, there was no pCT response with the F356A mutant, but this did not alter efficacy for sCT or hCT. The I371A mutant significantly enhanced efficacy for all peptides. The P360A mutant selectively reduced hCT efficacy, while the Y372A and M376A mutants selectively attenuated efficacy for pCT. No other mutants altered peptide-mediated IP1 efficacy (Fig. 11B, Fig. 12B, Table 6).

Within ECL2, efficacy was attenuated for all peptides at the Y299A mutant and enhanced at the H296A and I300A mutants, albeit that the H296A and I300A mutants did not achieve significance for hCT or pCT, respectively (Fig. 11C, Fig. 12C, Table 6). The C289A mutant selectively enhanced sCT efficacy, while no quantifiable response was observed for hCT at the R281A and W290A mutants, which did not alter sCT or pCT efficacy. Within ECL3, the mutant effects tended to be relatively peptide specific, although no measurable response was observed for any peptide at the I371A mutant (Fig. 11C, Fig. 12C, Table 6). sCT efficacy was least impacted with loss of efficacy at the Y372A mutant (that was also seen with hCT and pCT), and selective enhancement of efficacy at the F359A, this latter effect contrasted with the enhanced efficacy seen for hCT and pCT. The P360A and M376A mutants attenuated pCT and hCT efficacy, albeit that the M376A mutant did not achieve significance for hCT. The K366A mutant selectively enhanced hCT efficacy, while there was a selective loss of pCT efficacy with the D373A mutant, and selective enhancement of efficacy for the M367A and L368A mutants with this peptide (Fig. 11C, Fig. 12C, Table 6).

#### 3.4.2 Amylin and CGRP

Within ECL2 there was no quantifiable response for D287A for either amylin or CGRP, and no measurable response to CGRP at the C289A and W290A mutants, while the C289A mutant, along with N286A, had enhanced amylin efficacy (Fig. 11A, Fig. 12A, Table 6). Both amylin and CGRP efficacy were enhanced at the I300A mutant but there were no other significant effects for either peptide. Within ECL3, the F356A and I371A mutants enhanced efficacy for both peptides, while the Y372A mutant attenuated CGRP efficacy and abolished the response to amylin, but no other mutants impacted on amylin efficacy. For CGRP, loss of efficacy also occurred at the F359A, P360A, D373A and M376A mutants (Fig. 11A, Fig. 12A, Table 6).

Due to weak coupling of amylin and CGRP to IP1 signalling, effects of mutations on efficacy could not be determined.

As noted above for functional affinity data, as coupling of amylin and CGRP to pERK is relatively weak, many mutants had responses that could not be operationally quantified. Although some of these had selective effects on amylin or CGRP, in these cases the effects on affinity versus efficacy could not be separated.

Within ECL2, most mutants either had no quantifiable signalling or did not affect peptide efficacy. Lack of signalling occurred for both peptides at the D287A, C289A and W290A mutants. Signalling was not quantifiable for L291A and Y299A for CGRP, and attenuated for amylin, while amylin signalling was not quantifiable at the T295A mutant, with no effect on CGRP (Fig. 11C, Fig. 12C, Table 6).

Within ECL3, no quantifiable signalling for either peptide was observed for the P360A, D373A and I371A mutants. At the Y372A mutant, efficacy was abolished for amylin and attenuated for CGRP. Efficacy of both peptides was reduced at the W361A and M376A mutants. It was reduced for amylin and abolished for CGRP at the P363A mutant. In general, mutations to the membrane proximal segment of TM6/ECL3 had greater impact on CGRP efficacy, with either loss (V357A) or attenuation (V358A, F359A, R362A) of efficacy for CGRP with less pronounced effects amylin efficacy (Fig. 11C, Fig. 12C, Table 6).

## 4 Discussion

Recent structural biology breakthroughs for the CTR and GLP-1R have provided new understanding of class B GPCR peptide binding and receptor activation that includes reorganisation of the packing of loop residues, and major, conserved, conformational changes in TM6/ECL3/TM7 at the extracellular face of the receptor that are linked to outward movement of the intracellular face of TM6 to accommodate G protein binding [13], [14], [15]. However, these studies also revealed peptide/receptor specific differences in presentation of the peptide N-terminus to the core and their engagement with the receptor surface, in particular for ECLs 2 and 3. For the GLP-1R, these loops play an important role in peptide binding, efficacy and biased agonism [15], [16], [21]. Intriguingly, the role of ECLs 2 and 3 of the CTR was generally distinct when compared to the GLP-1R. This could in part be attributed to distinct effector coupling profiles exhibited by the two different receptors with the GLP-1R capable of coupling to both G proteins and β-arrestins [16], whereas CTR is unable to recruit the latter when activated by CT peptides [41].

### 4.1 CTR ECL2 plays a key role in conformational propagation linked to Gs/cAMP signalling that is distinct from that of GLP-1R ECL2

CTR stability, as indexed by cell surface expression, was highly sensitive to mutations in the core of ECL2 that formed an interconnected network but were located away from the principal binding site for sCT in the active structure. Moreover, mutation of this ECL2 network enhanced efficacy selectively for cAMP (all peptides) suggesting that the ECL2 destabilized state is linked to lowered barrier for Gs activation, despite decreased affinity of some mutations for peptides (lower pK_A_). Indeed, some mutants demonstrated higher Emax than WT receptor, despite low cell surface expression. However, there was limited correlation of the loss of cell surface expression with efficacy in other pathways, indicating that this ECL2 conformation is poorly linked to activation of other pathways for this receptor.

**Fig. 13:**
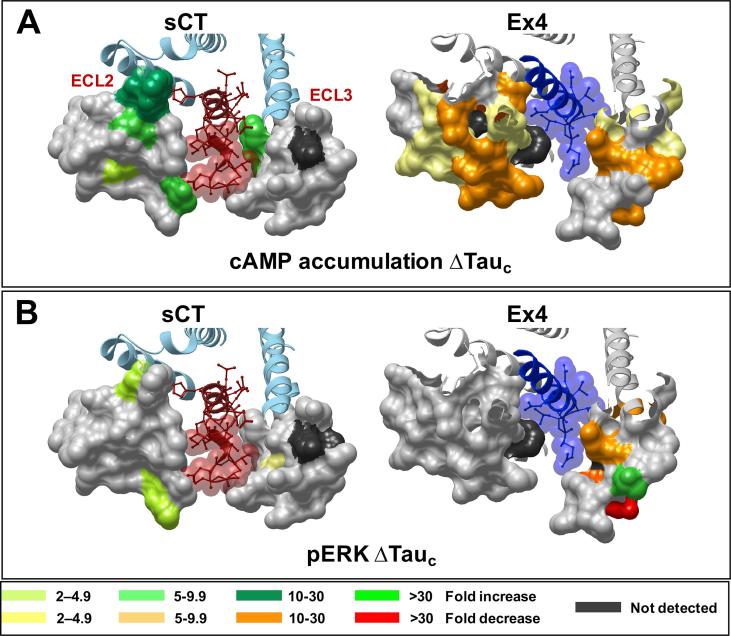
Comparison of the effect of alanine-scanning mutagenesis of ECL2 and ECL3 on cAMP (A) and pERK (B) efficacy of sCT at the CTR or exendin-4 at the GLP-1R. Top views of active state structures of sCT/hCTR/Gs or Ex-P5/hGLP-1R/Gs, with the receptor ECD and peptide C-terminus omitted for clarity. Mutations that significantly alter peptide efficacy are coloured according the magnitude of effect, with mutated amino acids without significant alteration to efficacy coloured grey. Efficacy data for exendin-4 (Ex4) are from Wootten et al., 2016, and are mapped onto the structure of exendin-P5/hGLP-1R/Gs (PDB: 6B3J). sCT is shown as dark red, with side chains in proximity to the ECLs displayed in x-stick, and residues 1–7 that are critical for receptor activation displayed in transparent cpk. Exendin-P5 is shown as dark blue, with side chains in proximity to the ECLs displayed in x-stick, and residues 1–8 that are important for receptor activation displayed in transparent cpk. (For interpretation of the references to colour in this figure legend, the reader is referred to the web version of this article.)

**Fig. 14:**
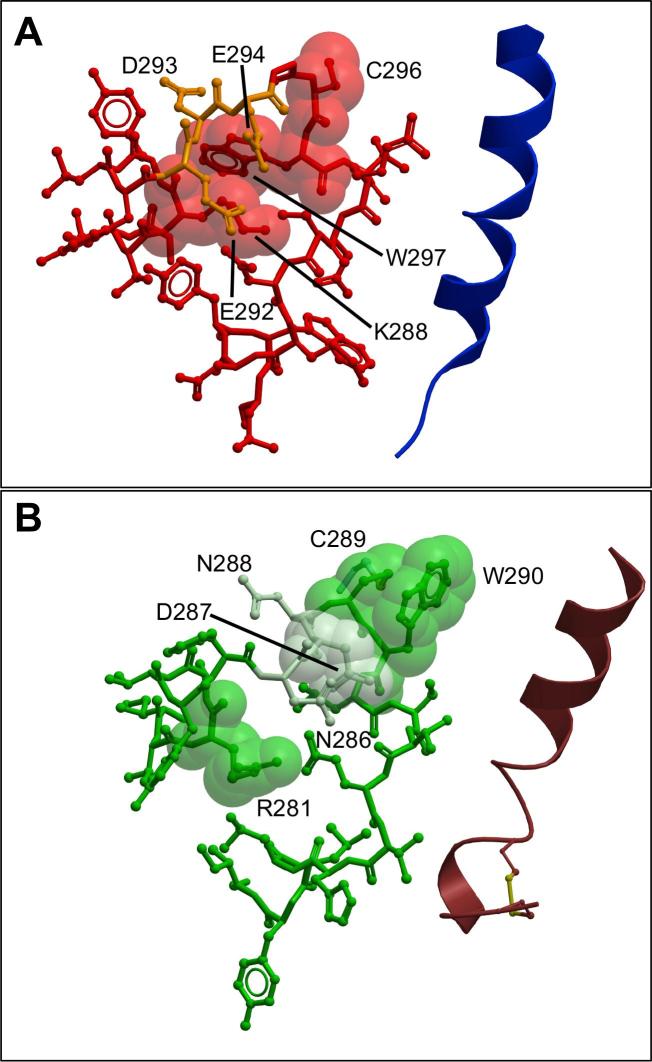
CTR and GLP-1R have distinct conformations of ECL2 that are differentially stabilised by conserved residues in the core of the loop. (A) GLP-1R displaying loop residues in red x-stick and the highly conserved C296, W297 and K288 residues displayed in transparent cpk representation. Shown in orange are GLP-1R residues in the conserved polar network E292, D293, and E294. (B) CTR displaying loop residues in green x-stick and the highly conserved C289, W290 and R281 residues displayed in transparent cpk representation. Shown in light green are the CTR residues in the equivalent conserved polar network N286, D287, and N288. Ribbon representations of the proximal amino acids of exendin-P5 (blue) or sCT (dark red) are also displayed. (For interpretation of the references to colour in this figure legend, the reader is referred to the web version of this article.)

**Fig. 15:**
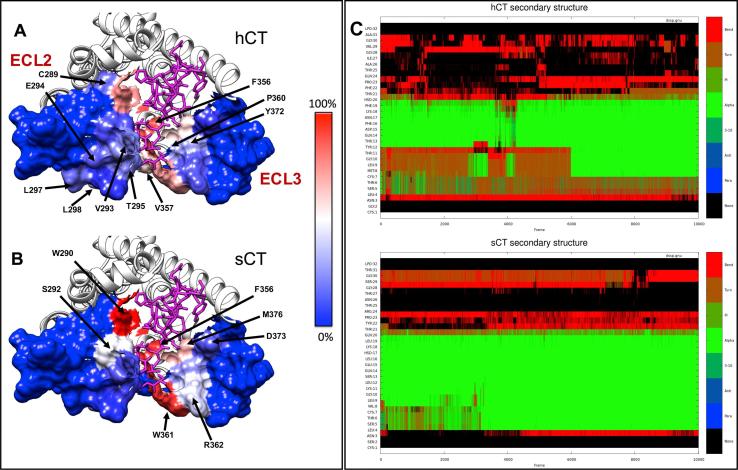
Molecular dynamic simulations of sCT and hCT peptide-receptor interaction. CTR/CT contacts identified during 1 μs MD simulations are plotted on the CTR molecular surface. The CT residues least engaged by the receptor (0% contact) are coloured blue, while residues most engaged by the receptor (100% contact) are coloured red. (A) hCT (magenta), (B) sCT (magenta). (C) Upper panel; secondary structure of hCT during the 1 μs MD simulation of hCT bound to CTR, as determined using VMD. Lower panel; secondary structure of sCT during the 1 μs MD simulation of sCT bound to CTR, as determined using VMD. (For interpretation of the references to colour in this figure legend, the reader is referred to the web version of this article.)

This segment of ECL2 contains a number of residues that are very highly conserved across the CTR and GLP-1R (and indeed all class B GPCRs), including R281/K288 (CTR/GLP-1R amino acids and residue number, respectively), C289/C296, W290/W297 and an polar/acidic motif between these residues N286,D287,N288 (CTR) and E292,D293,E294 (GLP-1R) [13], [14], [15]. Despite this, these residues are differentially important in receptor activation between those receptors. With minor exception, GLP-1R expression/stability was not markedly affected by mutation for any of the ECLs, including ECL2, however, ECL2 was broadly required for both Gs- (cAMP) and Gq- (iCa2+) mediated signalling with mutation decreasing peptide efficacy [16], [21]. This contrasts with both the enhancement of cAMP efficacy for CT peptides, and the very limited importance of ECL2 in CT efficacy for IP1 signalling (Fig. 11, Fig. 12, Fig. 13A). Comparison of the Gs complexed structures of the two receptors provides some potential insight into why these differences may occur, in particular, there are marked differences in positioning of W290/297 and the packing interactions of conserved residues around this residue. In the GLP-1R, W297 is completely flipped and buried within the core of the loop and this conformation is stabilized by K288, with the acidic/polar residues forming additional interactions that stabilize this conformation (Fig. 14). In contrast, the aromatic functional group of W290 in the CTR remains oriented towards the receptor core with extensive interactions observed with sCT and hCT that are stable in MD simulations (Fig. 15A, Table 7); D287 packs tightly with W290 and C289, while R281 forms alternate interactions to stabilize the loop conformation (Fig. 14B). The two receptors have very distinct preferred orientations of the N-terminal ECD and the peptide ligands enter the receptor core at different angles, with GLP-1/ExP5 closer to ECL2 such that their entry may require the major reorientation of W297 [15]. In this vein, it is interesting to note that while alanine mutations of C296 and W297 dramatically diminished GLP-1 and exendin-4 binding, they did not alter oxyntomodulin affinity [21], and it is possible that this peptide engages the receptor core in a manner more similar to that observed for sCT. For ECL2, CGRP and amylin were generally less affected by mutation and efficacy effects were either unmeasurable or only found in a subset of those with altered efficacy for CT peptides.

**Table 7:** Interactions between either hCT or sCT and the CTR in MD simulations of bound peptide.

CTR Residue	Interaction	hCT	sCT
I279	CTR H bonds	/	/
	CTR Contacts	/	/
T280	CTR H bonds	/	/
	CTR Contacts	/	/
R281	CTR H bonds	/	/
	CTR Contacts	/	/
V283	CTR H bonds	/	/
	CTR Contacts	/	/
Y284	CTR H bonds	/	/
	CTR Contacts	/	/
F285	CTR H bonds	/	/
	CTR Contacts	/	/
N286	CTR H bonds	/	/
	CTR Contacts	/	/
D287	CTR H bonds	/	/
	CTR Contacts	/	/
N288	CTR H bonds	/	/
	CTR Contacts	/	/
C289	CTR H bonds	/	/
	CTR Contacts	Q14 34.4%	/
W290	CTR H bonds	/	H17 3.0%C1 2.5% (bb)
	CTR Contacts	Q14 57.0%	S13 73.8%
		N17 54.7%	Q14 60.0%
		T13 30.9%	H17 59.0%
		K18 25.8%	G10 24.2%
L291	CTR H bonds	K18 2.3% (bs)	/
	CTR Contacts	Q14 54.9% T11 34.0%	/
S292	CTR H bonds	/	Q14 9.9%
	CTR Contacts	/	Q14 49.8% H17 28.0%
V293	CTR H bonds	/	/
	CTR Contacts	Q14 27.1%	Q14 21.8%
E294	CTR H bonds	C1 8.7% (sb)	/
	CTR Contacts	/	/
T295	CTR H bonds	/	/
	CTR Contacts	G2 22.5%	/
H296	CTR H bonds	/	/
	CTR Contacts	/	/
L297	CTR H bonds	/	/
	CTR Contacts	N3 26.2%	/
L298	CTR H bonds	N3 9.5% (bs)	/
	CTR Contacts	N3 62.5% T6 27.8%	N3 25.4%
Y299	CTR H bonds	/	/
	CTR Contacts	N3 47.8%	S2 29.5%
I300	CTR H bonds	/	/
	CTR Contacts	/	/
F356	CTR H bonds	/	S5 5.1% (bs)
	CTR Contacts	M8 74.1% S5 54.0% L4 37.1% L9 26.1%	L4 92.1% S5 77.4% V8 32.4%
V357	CTR H bonds	/	/
	CTR Contacts	L4 72.1% S5 41.7%	/
V358	CTR H bonds	/	/
	CTR Contacts	/	/
F359	CTR H bonds	/	/
	CTR Contacts	M8 42.8%	L4 60.6%
P360	CTR H bonds	/	/
	CTR Contacts	L4 67.2% M8 60.5% C7 41.8%%	L4 89.6% C1 26.3%
W361	CTR H bonds	/	/
	CTR Contacts	C1 37.9% C7 34.3% L4 28.6% T11 25.9%	L4 58.4% C1 43.2% C7 34.9% K11 31.8% V8 29.8% S2 26.8%
R362	CTR H bonds	/	/
	CTR Contacts	C1 20.3%	K11 60.2%
P363	CTR H bonds	/	/
	CTR Contacts	/	/
S364	CTR H bonds	/	/
	CTR Contacts	/	/
N365	CTR H bonds	/	/
	CTR Contacts	/	/
K366	CTR H bonds	/	/
	CTR Contacts	/	/
M367	CTR H bonds	/	/
	CTR Contacts	/	/
L368	CTR H bonds	/	/
	CTR Contacts	/	/
G369	CTR H bonds	/	/
	CTR Contacts	/	/
K370	CTR H bonds	/	/
	CTR Contacts	/	/
I371	CTR H bonds	/	/
	CTR Contacts	/	/
Y372	CTR H bonds	/	/
	CTR Contacts	M8 36.8%	L4 45.4%
D373	CTR H bonds	C1 6.8% (sb)	K11 13.0%
	CTR Contacts	/	K11 27.8%
Y374	CTR H bonds	/	/
	CTR Contacts	/	/
V375	CTR H bonds	/	/
	CTR Contacts	/	/
M376	CTR H bonds	/	/
	CTR Contacts	M8 77.2%	L4 82.2% V8 49.7%

Hydrogen bonds and generic contacts established between the CTR and both the human calcitonin (hCT) and salmon calcitonin (sCT), during 2 μs of MD simulations (percentages are referred to the total number of frames). (sb): hydrogen bond involving a CTR side chain and the backbone of the hCT or sCT; (bs) hydrogen bond involving a CTR backbone atom and a hCT or sCT side chain. If not specified, the hydrogen bond refers to both the CTR and the hCT or sCT side chains. Only values higher than 2% for hydrogen bonds and 20% for generic contacts are reported*.*

The CTR shares greatest homology with the calcitonin receptor-like receptor (CLR), including strong conservation of residues within ECL2. Unlike CTR, CLR requires RAMP interaction for functional cell surface expression and to form CGRP (CGRP_1_, CLR/RAMP1) or adrenomedullin (AM_1_, CLR/RAMP2; AM_2_, CLR/RAMP3) receptors. In contrast to the CTR, CLR cell surface expression was not greatly impacted by alanine mutation of ECL2 residues [42], [43]. However, there were similarities in the impact of mutation of conserved residues on cAMP pK_A_ (CTR) or cAMP potency (CLR/RAMP receptors). This included reductions in CGRP and adrenomedullin potency, for their respective receptors, for R274A (R281A, in CTR), D280A (D287A), C282A (C289A), W283A (W297A), and I284A (L291A) [42] that paralleled the losses in functional affinity seen with hCT and pCT, although this could be RAMP-dependent for the adrenomedullin receptors [43].

Within ECL2, amino acids proximal to the peptide in the sCT/CTR/Gs structure (W290-T295; L297,L298) tended to display peptide and/or pathway selective effects. These likely form dynamic and differential interactions with key polar residues of the peptides (S^2,sCT/pCT^/G^2,hCT^, N^3^, T^6^, Q^14,sCT/hCT^,R^14,pCT^, H^17,sCT^/N^17,hCT/pCT^) to influence peptide binding and signalling (Fig. 15A; Table 7; Supplementary Movie 1

Movie 1Interactions between hCT (left hand panel) or sCT (right hand panel) with the CTR during 1 μs MD simulation. Interactions between hCT and the CTR are less stable than those of sCT and the receptor. As a consequence, ECL2 in the hCT-bound CTR undergoes more dynamic conformational sampling.
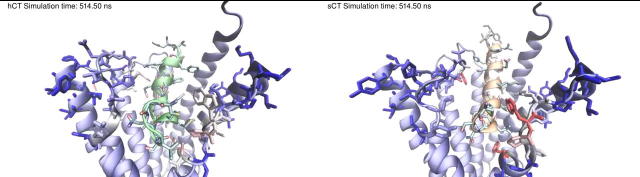

). Of note, T288A, L290A, and L291A of CLR, in a RAMP-dependent manner, also attenuated adrenomedullin (T288A) or CGRP (T288A,L290A,L291A) cAMP potency [42], [43]. Intriguingly, comparison of 1 μs MD simulations of hCT and sCT bound to the CTR indicated that ECL2 was more conformationally dynamic when the receptor was bound to hCT (Supplementary Movie 1), and this may also contribute to the differential effects of mutation between CT peptides.

### 4.2 ECL3 is a gateway for ligand, receptor and pathway specific modulation of class B GPCR function

Across the available active CTR and GLP-1R structures, the largest difference in the receptor core was the angle of tilt of TM6, and to a lesser extent TM7, and the interconnecting conformation of ECL3, with the CTR exhibiting the greatest outward movement of this domain [13], [14], [15]. Nonetheless, this was also the region of greatest divergence between the structures of GLP-1/GLP-1R/Gs and the G protein-biased analogue complex, ExP5/GLP-1R/Gs, indicating that peptide interactions with ECL3 play a critical role in differential modes of receptor activation [15]. Indeed, comparison of the effect of mutation in ECL3 across multiple peptides and pathways, and between the CTR and GLP-1Rs, revealed significant diversity in how ECL3 was engaged and contributed to peptide binding and propagation of conformational change linked to efficacy (Fig. 13). Interestingly, mutations that altered GLP-1R mediated Ca^2+^ (Gq) and pERK primarily clustered within ECL2 and ECL3 respectively, whereas CTR mutations that altered IP1 (Gq) and pERK displayed similar clustering, predominantly within ECL3 that distinct from those required for cAMP. pERK can be activated downstream of multiple effector proteins and is often a composite of many divergent signalling pathways [16], [44]. While pERK1/2 mediated by the GLP-1R is a composite of both G protein and β-arrestin signalling [16], the CTR is unable to recruit β-arrestins, suggesting that the pERK response is likely to be predominantly G protein mediated [41]. While the CTR can couple to multiple different G proteins, similar clustering in our of residues important for IP1 and pERK in our mutational analysis suggests that CTR mediated pERK, at least in part, may be downstream of Gq coupling, although further experimental data will be required to confirm this.

Despite the diversity in how CTR and GLP-1Rs engage ECL3 to promote signalling, there were clear patterns with respect to clustering of residues that were functionally important, particularly around the TM6 and TM7 proximal segments of ECL3 that were located with 5 Å of the sCT ligand, and the network of residues within the loop that stabilized these interactions. For hCT and pCT, the sequence of residues at the apex of the TM extension (K366-G369) was important, in a peptide specific manner, for IP1 or pERK signalling, indicating that secondary structure in this segment of ECL3 contributes to propagation of conformation linked to these pathways for the less well coupled peptides. Of particular note were the clear distinctions in the patterns of important residues for pCT versus other CT peptides, and CGRP across all peptides suggesting that these peptides have different modes of ligand engagement with the receptor relative to the other peptides.

Uniquely among class B GPCR peptide ligands, the CT-family peptides contain an N-terminal disulphide bridge between residues 1 and 7 (2 and 7 for CGRP and adrenomedullin), with a consequent bulky loop structure that contrasts to the linearly extended GLP-1R peptides observed in the active structures. This loop is oriented toward ECL3 and the larger outward movement of this domain is required to accommodate the peptide N-terminus. However, the peptides are predicted to make relatively weak (non-polar) and transient interactions with ECL3 (Fig. 15A; Table 7), and this is consistent with the high mobility of TM6/ECL3 in the sCT/CTR/Gs structure that could not be resolved at high resolution [13]. Linear analogues of sCT maintain high affinity and potency in cAMP signalling, whereas equivalent analogues of hCT have attenuated potency [9], [45], [46]. sCT has higher helical secondary structure propensity in the mid-region of the peptide, compared to hCT [9], [47] that likely constrains the location of the N-terminus to maintain interactions, whereas the additional constraints imposed by the disulphide bridge are required to facilitate interactions for hCT. Greater secondary structure of sCT versus hCT is seen in MD simulations of bound peptides (Fig. 15C) and this contributes to predicted differences in peptide-receptor interactions for these two peptides (Fig. 15B; Table 7, Supplementary Movie 1).

Intriguingly, comparison of the ExP5, and the GLP-1, receptor complexes, revealed distinct positioning of peptides (including minor difference in the relative orientation of the ECD) that likely contributes to engagement with ECL3/TM6 and TM7, and this had implications for ligand-dependent G protein conformations and cAMP signalling efficacy [14], [15]. Amongst the cryo-EM structures, the GLP-1R complexes exhibited a single major conformation of the ECD relative to the receptor core, while the sCT/CTR/Gs complex contained multiple conformations of the ECD that were discernible at lower resolution [13]. CT family peptides have a relatively unstructured, and more flexible C-terminus than GLP-1 and related peptides [13], [48], and it is likely that there would be greater potential for different CT peptides to have altered orientation within the receptor core, relative to sCT, as would be predicted from MD simulations (Fig. 15C). This would be consistent with the differential impact of ECL3 mutation on sCT versus hCT and pCT, and between hCT and pCT (eg. for pK_A_ in IP1 and pERK, and efficacy for cAMP and pERK).

Though it is difficult to draw direct comparisons, it is intriguing that select mutation of residues in ECL3 differentially affected potency/functional affinity of GLP-1 relative to ExP5 [15], and that this was also seen for sCT versus hCT. Like hCT and sCT that have altered Gs-mediated efficacy linked to different ligand-induced conformations of Gs [11], higher efficacy was observed for ExP5 (relative to affinity) compared to GLP-1, and this was associated with lower population based assessment of max conformational change (hCT, ExP5 versus sCT, Ex4/GLP-1, respectively). This provides the first evidence of parallels in the mode of control of efficacy within relatively divergent class B subfamily members, albeit that there is still much to understand at a mechanistic level.

Amylin and CGRP are low affinity and potency agonists of CTR but are high potency ligands of CTR/RAMP complexes [8]. While this low potency limited analysis for poorly expressed CTR mutants, the impact of mutation on amylin was generally consistent with observations for CT peptides, especially sCT. In contrast, CGRP had a unique profile, particularly with respect to residues within ECL3 and proximal to TM6 (F356A-R362A), indicative of a non-canonical spectrum of interaction with this receptor segment. Of note, there were additional CGRP selective effects for mutations to CTR residues Y372 and D373 that could make direct peptide interactions, and M367 that packs between F356 and F359. Data from the related secretin receptor, has implicated TM6/TM7 as the principal membrane interface for RAMP interaction [49], and this is broadly consistent with the data for CLR [13], albeit that the authors of this latter work reached a different conclusion. While CTR and CLR interactions are more complex, with additional interaction between RAMP and receptor ECDs that contribute to altered peptide affinities [48], [49], [50], a TM6 interface would be consistent with the key role of ECL3 in biased signalling, and with the predicted impact of RAMP interactions on the conformation of ECL3 for adrenomedullin receptors [43]. RAMPs alter signalling profiles for multiple class B GPCRs, including glucagon, VPAC1 and CT receptors [50], [51], [52], [53], [54], [55], even where altered binding phenotypes are not observed. The nature of the RAMP-induced enhancement of CGRP binding and potency at the CTR is particularly complex, with RAMP chimera and truncation studies indicative of a direct role of the short RAMP C-terminus in G protein engagement that is critical for CGRP potency [56], [57], and this may also allosterically regulate CGRP engagement with ECL3. ECL3 was also important for cAMP signalling of CGRP or adrenomedullin at CLR/RAMP complexed receptors. Similar to observations for CT peptides at the CTR, both common and peptide specific effects were observed [58], [59], [60], confirming the dynamic role that ECL3 plays in this receptor subfamily. However, as the signalling data were not separated into the derivative effects on affinity and efficacy, it is difficult to make specific comparisons. Understanding the impact of RAMPs on CTR binding and activation by peptides will require additional structures and mutants of RAMP-complexed receptors.

Integration of alanine scanning analysis of the critically important ECLs 2 and 3, on peptide function, with new insights available from novel peptide-bound, active state, G protein complexed class B receptors, has revealed marked diversity in mechanisms of peptide engagement and receptor activation between the CTR and GLP-1R. A significant contributor appears to be the orientation of peptides within the receptor core, and this will be influenced by relative orientation of the ECD that engages the peptide C-terminus. While both domains play important functional roles, ECL3 appears to be a hotspot for distinct ligand and pathway specific effects, and this has implications for the future design of biased agonists of class B GPCRs.

## References

[1] Wootten D., Miller L.J., Koole C., Christopoulos A., Sexton P.M. (2017). Allostery and biased agonism at Class B G protein-coupled receptors. Chem. Rev..

[2] D.M. Findlay, P.M. Sexton, T.J. Martin, Calcitonin, in: J.L. Jameson, L.J. De Groot, D.M. de Kretser, L.C. Giudice, A.B. Grossman, S. Melmed, J.T. Potts Jr., G.C. Weir (Eds.), Endocrinology: Adult & Pediatric, Vols 1 and 2, 7th ed. Philadelphia PA USA: Elsevier, pp. 1004–1017 14 p. (2015).

[3] Minhas P.S., Virdi J.K. (2017). Hypercalcemia in inpatient setting: Diagnostic approach and management. Curr. Emerg. Hosp. Med. Rep..

[4] Langston A.L., Ralston S.H. (2004). Management of Paget’s disease of bone. Rheumatology (Oxford).

[5] Cosman F., de Beur S.J., LeBoff M.S., Lewiecki E.M., Tanner B., Randall S., Lindsay R., National Osteoporosis Foundation (2014). Clinician’s guide to prevention and treatment of osteoporosis. Osteoporos. Int..

[6] Kuestner R.E., Elrod R., Grant F.J., Hagen F.S., Kuijper J.L., Matthewes S.L., Sheppard P.O., Stroop S.D., Thompson D.L., Whitmore T., Findlay D.M., Houssami S., Sexton P.M., Moore E.E. (1994). Cloning and characterization of an abundant subtype of the human calcitonin receptor. Mol. Pharmacol..

[7] Poyner D.R., Sexton P.M., Marshall I., Quirion R., Kangawa K., Born W., Fischer J.A., Muff R., Smith D.M., Foord S.M. (2002). The mammalian CGRP, adrenomedullin, amylin and calcitonin receptors. Pharmacol. Rev..

[8] Hay D.L., Pioszak A.A. (2016). Receptor activity-modifying proteins (RAMPs): new insights and roles. Annu. Rev. Pharmacol. Toxicol..

[9] Hilton J.M., Dowton M., Houssami S., Sexton P.M. (2000). Identification of key components in the irreversibility of salmon calcitonin binding to calcitonin receptors. J. Endocrinol..

[10] Andreassen K.V., Hjuler S.T., Furness S.G., Sexton P.M., Christopoulos A., Nosjean O., Karsdal M.A., Henriksen K. (2014). Prolonged calcitonin receptor signaling by salmon, but not human calcitonin, reveals ligand bias. PLoS One.

[11] Furness S.G.B., Liang Y.L., Nowell C.J., Halls M.L., Wookey P.J., Dal Maso E., Inoue A., Christopoulos A., Wootten D., Sexton P.M. (2016). Ligand-dependent modulation of G protein conformation alters drug efficacy. Cell.

[12] de Graaf C., Song G., Cao C., Zhao Q., Wang M.-W., Wu B., Stevens R.C. (2017). Extending the structural view of Class B GPCRs. Trends Biochem. Sci..

[13] Y.L. Liang, M. Khoshouei, M. Radjainia, Y. Zhang, A. Glukhova, J. Tarrasch, D.M. Thal, S.G.B. Furness, G. Christopoulos, T. Coudrat, R. Danev, W. Baumeister, L.J. Miller.

[14] Christopoulos A., Kobilka B.K., Wootten D., Skiniotis G., Sexton P.M. (2017). Phase-plate cryo-EM structure of a class B GPCR-G-protein complex. Nature.

[15] Zhang Y., Sun B., Feng D., Hu H., Chu M., Qu Q., Tarrasch J.T., Li S., Kobilka T.S., Kobilka B.K., Skiniotis G. (2017). Cryo-EM structure of the activated GLP-1 receptor in complex with a G protein. Nature.

[16] Liang Y.L., Khoshouei M., Glukhova A., Furness S.G.B., Zhao P., Clydesdale L., Koole C., Truong T.T., Thal D.M., Lei S., Radjainia M., Danev R., Baumeister W., Wang M.-W., Miller L.J., Christopoulos A., Sexton P.M., Wootten D. (2018). 3.3Å phase-plate cryo-EM structure of a biased agonist-bound human GLP-1 receptor-Gs complex. Nature.

[17] Wootten D., Reynolds C.A., Smith K.J., Mobarec J.C., Koole C., Savage E.E., Pabreja K., Simms J., Sridhar R., Furness S.G.B., Liu M., Thompson P.E., Miller L.J., Christopoulos A., Sexton P.M. (2016). The extracellular surface of the GLP-1 receptor is a molecular trigger for biased agonism. Cell.

[18] Dong M., Lam P.C., Orry A., Sexton P.M., Christopoulos A., Abagyan R., Miller L.J. (2016). Use of cysteine trapping to map spatial approximations between residues contributing to the helix N-capping motif of secretin and distinct residues within each of the extracellular loops of its receptor. J. Biol. Chem..

[19] Dong M., Koole C., Wootten D., Sexton P.M., Miller L.J. (2014). Structural and functional insights into the juxtamembranous amino-terminal tail and extracellular loop regions of class B GPCRs. Br. J. Pharmacol..

[20] Woolley M.J., Conner A.C. (2017). Understanding the common themes and diverse roles of the second extracellular loop (ECL2) of the GPCR super-family. Mol. Cell. Endocrinol..

[21] Weaver R.E., Mobarec J.C., Wigglesworth M.J., Reynolds C.A., Donnelly D. (2017). High affinity binding of the peptide agonist TIP-39 to the parathyroid hormone 2 (PTH_2_ receptor requires the hydroxyl group of Tyr-318 on transmembrane helix 5. Biochem. Pharmacol..

[22] Koole C., Wootten D., Simms J., Valant C., Sridhar R., Woodman O.L., Miller L.J., Summers R.J., Christopoulos A., Sexton P.M. (2010). Allosteric ligands of the glucagon-like peptide 1 receptor (GLP-1R) differentially modulate endogenous and exogenous peptide responses in a pathway-selective manner: implications for drug screening. Mol. Pharmacol..

[23] Koole C., Wootten D., Simms J., Miller L.J., Christopoulos A., Sexton P.M. (2012). Second extracellular loop of human glucagon-like peptide-1 receptor (GLP-1R) has a critical role in GLP-1 peptide binding and receptor activation. J. Biol. Chem..

[24] Johansson E., Hansen J.L., Hansen A.M.K., Shaw A.C., Becker P., Schaffer L., Reedtz-Runge S. (2016). Type II Turn of receptor-bound salmon calcitonin revealed by X-ray crystallography. J. Biol. Chem..

[25] Eswar N., Eramian D., Webb B., Shen M.Y., Sali A. (2008). Protein structure modeling with MODELLER. Methods Mol. Biol..

[26] Baldwin J.M., Schertler G.F., Unger V.M. (1997). An alpha-carbon template for the transmembrane helices in the rhodopsin family of G-protein-coupled receptors. J. Mol. Biol..

[27] Hamelryck T. (2005). An amino acid has two sides: a new 2D measure provides a different view of solvent exposure. Proteins.

[28] Doerr S., Harvey M.J., Noé F., De Fabritiis G. (2016). HTMD: high-throughput molecular dynamics for molecular discovery. J. Chem. Theory Comput..

[29] Dolinsky T.J., Nielsen J.E., McCammon J.A., Baker N.A. (2004). PDB2PQR: an automated pipeline for the setup of Poisson-Boltzmann electrostatics calculations. Nucl. Acids Res..

[30] Li H., Robertson A.D., Jensen J.H. (2005). Very fast empirical prediction and rationalization of protein pKa values. Proteins.

[31] Sommer B. (2013). Membrane packing problems: a short review on computational membrane modeling methods and tools. Comput. Struct. Biotechnol. J..

[32] Lomize M.A., Lomize A.L., Pogozheva I.D., Mosberg H.I. (2006). OPM: orientations of proteins in membranes database. Bioinformatics.

[33] Jorgensen W.L., Chandrasekhar J., Madura J.D., Impey R.W., Klein M.L. (1983). Comparison of simple potential functions for simulating liquid water. J. Chem. Phys..

[34] Huang J., MacKerell A.D. (2013). CHARMM36 all-atom additive protein force field: validation based on comparison to NMR data. J. Comput. Chem..

[35] Harvey M.J., Giupponi G., Fabritiis G.D. (2009). ACEMD: accelerating biomolecular dynamics in the microsecond time scale. J. Chem. Theory Comput..

[36] Berendsen H.J.C., Postma J.P.M., van Gunsteren W.F., DiNola A., Haak J.R. (1984). Molecular dynamics with coupling to an external bath. J. Chem. Phys..

[37] Loncharich R.J., Brooks B.R., Pastor R.W. (1992). Langevin dynamics of peptides: the frictional dependence of isomerization rates of N-acetylalanyl-N′-methylamide. Biopolymers.

[38] Krautler V., van Gunsteren W.F., Hanenberger P.H. (2001). A fast SHAKE algorithm to solve distance constraint equations for small molecules in molecular dynamics simulations. J. Comput. Chem..

[39] Essmann U., Perera L., Berkowitz M.L., Darden T., Lee H., Pedersen L.G. (1995). A smooth particle mesh Ewald method. J. Chem. Phys..

[40] Humphrey W., Dalke A., Schulten K. (1996). VMD: visual molecular dynamics. J. Mol. Graph.

[41] Dal Maso E., Just R., Hick C., Christopoulos A., Sexton P.M., Wootten D., Furness S.G.B. (2017). Characterisation and signalling and regulation of common calcitonin receptor splice variants and polymorphisms. Biochem. Pharmacol..

[42] Black J.W., Leff P. (1983). Operational models of pharmacological agonism. Proc. R. Soc. Lond. B Biol. Sci..

[43] Woolley M.J., Watkins H.A., Taddese B., Karakullukcu Z.G., Barwell J., Smith K.J., Hay D.L., Poyner D.R., Reynolds C.A., Conner A.C. (2013). The role of ECL2 in CGRP receptor activation: a combined modelling and experimental approach. J. R. Soc. Interface.

[44] Belcheva M.M., Coscia C.J. (2002). Diversity of G protein coupled receptor signalling pathways to ERK/MAP kinase. Neurosignals.

[45] Watkins H.A., Chakravarthy M., Abhayawardana R.S., Gingell J.J., Garelja M., Pardamwar M., McElhinney J.M., Lathbridge A., Constantine A., Harris P.W., Yuen T.Y., Brimble M.A., Barwell J., Poyner D.R., Woolley M.J., Conner A.C., Pioszak A.A., Reynolds C.A., Hay D.L. (2016). Receptor activity-modifying proteins 2 and 3 generate adrenomedullin receptor subtypes with distinct molecular properties. J. Biol. Chem..

[46] Orlowski R.C., Epand R.M., Stafford A.R. (1987). Biologically potent analogues of salmon calcitonin which do not contain an N-terminal disulfide-bridged ring structure. Eur. J. Biochem..

[47] Feyen J.H., Cardinaux F., Gamse R., Bruns C., Azria M., Trechsel U. (1992). N-terminal truncation of salmon calcitonin leads to calcitonin antagonists. Structure activity relationship of N-terminally truncated salmon calcitonin fragments in vitro and in vivo. Biochem. Biophys. Res. Commun..

[48] Epand R.M., Epand R.F., Orlowski R.C. (1988). Biologically active calcitonin analogs which have minimal interactions with phospholipids. Biochem. Biophys. Res. Commun..

[49] Booe J.M., Walker C.S., Barwell J., Kuteyi G., Simms J., Jamaluddin M.A., Warner M.L., Bill R.M., Harris P.W., Brimble M.A., Poyner D.R., Hay D.L., Pioszak A.A. (2015). Structural basis for receptor activity-modifying protein-dependent selective peptide recognition by a G protein-coupled receptor. Mol. Cell.

[50] Harikumar K.G., Simms J., Christopoulos G., Sexton P.M., Miller L.J. (2009). Molecular basis of association of receptor activity-modifying protein 3 with the family B G protein-coupled secretin receptor. Biochemistry.

[51] Kuwasako K., Kitamura K., Nagata S., Kato J. (2009). Flow cytometric analysis of the calcitonin receptor-like receptor domains responsible for cell-surface translocation of receptor activity-modifying proteins. Biochem. Biophys. Res. Commun..

[52] Gingell J., Simms J., Barwell J., Poyner D.R., Watkins H.A., Pioszak A.A., Sexton P.M., Hay D.L. (2016). An allosteric role for receptor activity-modifying proteins in defining GPCR pharmacology. Cell Discov..

[53] Christopoulos A., Christopoulos G., Morfis M., Udawela M., Laburthe M., Couvineau A., Kuwasako K., Tilakaratne N., Sexton P.M. (2003). Novel receptor partners and function of receptor activity-modifying proteins. J. Biol. Chem..

[54] Morfis M., Tilakaratne N., Furness S.G., Christopoulos G., Werry T.D., Christopoulos A., Sexton P.M. (2008). Receptor activity-modifying proteins differentially modulate the G protein-coupling efficiency of amylin receptors. Endocrinology.

[55] Weston C., Lu J., Li N., Barkan K., Richards G.O., Roberts D.J., Skerry T.M., Poyner D., Pardamwar M., Reynolds C.A., Dowell S.J., Willars G.B., Ladds G. (2015). Modulation of glucagon receptor pharmacology by receptor activity-modifying protein-2 (RAMP2). J. Biol. Chem..

[56] Weston C., Winfield I., Harris M., Hodgson R., Shah A., Dowell S.J., Mobarec J.C., Woodlock D.A., Reynolds C.A., Poyner D.R., Watkins H.A., Ladds G. (2016). Receptor activity-modifying protein-directed G protein signaling specificity for the calcitonin gene-related peptide family of receptors. J. Biol. Chem..

[57] Udawela M., Christopoulos G., Morfis M., Christopoulos A., Ye S., Tilakaratne N., Sexton P.M. (2006). A critical role for the short intracellular C terminus in receptor activity-modifying protein function. Mol. Pharmacol..

[58] Udawela M., Christopoulos G., Tilakaratne N., Christopoulos A., Albiston A., Sexton P.M. (2006). Distinct receptor activity-modifying protein domains differentially modulate interaction with calcitonin receptors. Mol. Pharmacol..

[59] Barwell J., Conner A., Poyner D.R. (2011). Extracellular loops 1 and 3 and their associated transmembrane regions of the calcitonin receptor-like receptor are needed for CGRP receptor function. Biochim. Biophys. Acta.

[60] Kuwasako K., Hay D.L., Nagata S., Hikosaka T., Kitamura K., Kato J. (2012). The third extracellular loop of the human calcitonin receptor-like receptor is crucial for the activation of adrenomedullin signalling. Br. J. Pharmacol..

